# The slowing pace of life expectancy gains since 1950

**DOI:** 10.1186/s12889-018-5058-9

**Published:** 2018-01-17

**Authors:** Carolina Cardona, David Bishai

**Affiliations:** Department of Population, Family and Reproductive Health, Johns Hopkins Bloomberg School of Public Health, 615 N. Wolfe Street, Baltimore, MD 21205 USA

**Keywords:** Life expectancy at birth, Mortality reduction, International demographic trends, Cross country regression

## Abstract

**Background:**

New technological breakthroughs in biomedicine should have made it easier for countries to improve life expectancy at birth (LEB). This paper measures the pace of improvement in the decadal gains of LEB, for the last 60-years adjusting for each country’s starting point of LEB.

**Methods:**

LEB increases over the next 10-years for 139 countries between 1950 and 2009 were regressed on LEB, GDP, total fertility rate, population density, CO2 emissions, and HIV prevalence using country-specific fixed effects and time-dummies. Analysis grouped countries into one-of-four strata: LEB < 51, 51 ≤ LEB < 61, 61 ≤ LEB < 71, and LEB ≥ 71.

**Results:**

The rate of increase of LEB has fallen consistently since 1950 across all strata. Results hold in unadjusted analysis and in the regression-adjusted analysis. LEB decadal gains fell from 4.80 (IQR: 2.98–6.20) years in the 1950s to 2.39 (IQR:1.80–2.80) years in the 2000s for the healthiest countries (LEB ≥ 71). For countries with the lowest LEB (LEB < 51), decadal gains fell from 7.38 (IQR:4.83–9.25) years in the 1950s to negative 6.82 (IQR: -12.95--1.05) years in the 2000s. Multivariate analysis controlling for HIV prevalence, GDP, and other covariates shows a negative effect of time on LEB decadal gains among all strata.

**Conclusions:**

Contrary to the expectation that advances in health technology and spending would hasten improvements in LEB, we found that the pace-of-growth of LEB has slowed around the world.

**Electronic supplementary material:**

The online version of this article (10.1186/s12889-018-5058-9) contains supplementary material, which is available to authorized users.

## Background

New technological breakthroughs in biomedicine consume an increasing share of the global economy. Technology ought to enable production to become more efficient. In the production of population health, one would expect that the discovery of new vaccines, drugs, and surgeries would enable more rapid improvement of population health even if investments in health remained the same. In the last 60 years with both improved biomedical technology and the large increases in health care spending per capita one would expect to see progress in the rate at which a country with a low life expectancy of say 50 or 60 years could achieve improvements.

Multiple factors must work together to improve a country’s life expectancy at birth (LEB). Economic resources are a necessary, but not sufficient input into better population health [1, 2]. Scientific breakthroughs can help, but the world has abundant examples of poor populations who fail to access some of the lowest cost public health interventions like basic sanitation and vector control. People in lower income countries not only have lower levels of life expectancy than developed countries, but also live in poor health a high proportion of their lives [3].

Biological limits to the human lifespan imply that LEB gains will slow down as populations achieve higher and higher LEB [4]. Mortality improvements were linked with better policies that allowed advances in income, salubrity, education, sanitation and medicine [5]. These gains become harder to achieve as life expectancy becomes higher. So when the pace of improvement in life expectancy slows down one has to distinguish the role of ceiling effects from a slow down due to ineffective policy, misapplication of health technology or other factors.

The goal of this paper is to measure period effects on the rate at which life expectancy grows over time. Referring to the rate of change of LEB over time as “velocity” (dLEB/dt), we are concerned with asking whether the last 60 years have seen acceleration (d^2^LEB/dt^2^ > 0) or deceleration (d^2^LEB/dt^2^ < 0). In the 60 year trajectory of any single country it is trivial to show that there is deceleration as life expectancy approaches its upper limits. The non-trivial question is whether a typical country with a life expectancy of 50 years in 2010 had better future health prospects than a typical country with a life expectancy of 50 years in 1950. By stratifying countries according to their starting life expectancy in each period from 1950 to 2010 we can quantify how much global changes in living standards and biomedical technology have made it easier or harder to improve life expectancy.

The objective of this paper is to assess the pace of improvement in LEB in the last 60 years within comparable strata of life expectancy from low LEB (< 51) to high LEB (≥71). The analysis will try to explain changes in the pace of life expectancy improvement over time for each stratum.

## Methods

To adjust for a country’s starting point of LEB we divided countries into four strata according to their starting LEB: LEB < 51, 51 ≤ LEB < 61, 61 ≤ LEB < 71, and LEB ≥ 71. The analysis allowed each country to be reclassified into a new stratum whenever its life expectancy crossed a specific age cut, threshold. Over the 60-years most countries left a lower stratum to join a higher one. Many countries crossed 2–3 life expectancy strata, contributing country-years of observation in multiple strata. Table A-2-3 of the Additional file 1 lists countries by strata using a representative year for each decade, and Tables A-2-4 through A-2-7 show summary statistics by strata.

### Data

We obtained data for a set of 173 countries on life expectancy at birth and a set of key socioeconomic factors over a 60-year period, 1950–2009, all from publicly available databases. LEB data were from the Institute for Health Metrics and Evaluation (IHME) [6] for 1970–2009. Prior to 1970 LEB data were from: Human Mortality Database (HMD) [7–9], World Population Prospects (WPP) [10, 11], and Gapminder [12], see Table A-1-1 of the Additional file 1.

Socioeconomic factors identified in the literature as contributing to life expectancy improvement were: Population density and GDP per capita, as proxies for urbanization and economic growth; CO2 emissions, as a proxy for environmental quality. Total Fertility Rate (TFR) and HIV prevalence are included because of their known correlation with population health. Summary statistics of the total sample of countries are in Table 1, stratified by decade.

**Table 1:** Descriptive statistics by decade, total sample

Variables	Decade 1950–1959		Decade 1960–1969		Decade 1970–1979		Decade 1980–1989		Decade 1990–1999		Decade 2000–2009	
	Mean (SD)	Obs.	Mean (SD)	Obs.	Mean (SD)	Obs.	Mean (SD)	Obs.	Mean (SD)	Obs.	Mean (SD)	Obs.
LEB decade gain	9.68 (7.18)	1390	4.8 (2.21)	1390	3.72 (2.69)	1390	3.02 (2.31)	1390	1.48 (3.16)	1390	1.89 (3.67)	1390
Life Expectancy at Birth	53.54 (11.13)	1390	58.34 (10.23)	1390	62.06 (8.99)	1390	65.07 (8.53)	1390	66.55 (9.20)	1390	68.45 (9.58)	1390
GDP per capita, 2005, PPP $	5269 (4491)	569	5003 (5718)	968	6643 (7437)	1200	7912 (9160)	1207	9130 (11112)	1358	11,626 (14522)	1390
Population density per km^2^	72.37 (190.16)	1390	87.7 (251.94)	1390	102.36 (297.88)	1390	119.89 (354.34)	1390	141.5 (448.41)	1390	164.38 (556.94)	1390
CO2 in tons per capita	2.26 (4.23)	991	3.17 (7.88)	1148	4.46 (8.63)	1207	4.01 (5.92)	1210	4.23 (6.45)	1342	4.39 (6.39)	1383
Total Fertility Rate	5.24 (1.76)	1390	5.2 (1.89)	1390	4.78 (2.06)	1390	4.28 (2.06)	1390	3.62 (1.88)	1390	3.11 (1.70)	1390
HIV Prevalence	0 (0.00)	1390	0 (0.00)	1390	0.26 (0.76)	1390	0.87 (2.05)	1390	1.53 (3.59)	1390	2.08 (4.69)	1385

Population Density (per square km) was retrieved from the United Nations Population Division [13]. Real GDP per capita is measured at 2005 constant prices, and was retrieved from Penn World Table of the University of Pennsylvania [14].

Average CO2 emissions are measured in metric tons per person, and were retrieved from the Carbon Dioxide Information Analysis Center (CDIAC) [15]. A country’s level of carbon dioxide is a proxy of the greenness of an economy’s production processes. CO2 is included in the model to control for confounding time trends in air pollution, which has a known negative effect on health.

TFR data for 160 countries were retrieved from the United Nations Population Division [16]. For the 13 remaining countries data were pulled from different sources, see Data Sources of the Additional file 1. Estimated HIV prevalence data were obtained from the Joint United Nations Programme on HIV/AIDS (UNAIDS) [17]. The global HIV/AIDS pandemic had a high impact on the overall number of deaths worldwide. In some low income countries, an estimated of 1–4 years of life expectancy are lost due to HIV/AIDS in those older than 5 years [18]. For the years prior to 1970, it was assumed that HIV prevalence was virtually zero, since the disease was first acknowledged in 1981 [19]. Some countries lacked complete data on HIV between 1970 and 1989. For these countries, country-specific trends after 1990 were used to back-cast HIV rates into the prior period, see HIV Imputation – Table A-4-1 of the Additional file 1.

### Statistical methods

LEB decadal gains was used as the dependent variable, defined as the difference between LEB_t + 10_ and LEB_t_. Analysis was performed separately within each LEB strata to confine comparisons of countries with roughly equivalent LEB starting points.

For the regression-adjusted analysis, a country fixed effects model was selected. This model controls for unobservable time-invariant features of each country [20, 21]. The final model included the following 6 covariates – motivated by theory, past evidence, and data availability: LEB, GDP per capita, population density, CO2 emissions, TFR, and HIV prevalence. The model also included time dummies for each decade from 1950 - 1959 to 2000–2009.

Each covariate was converted to a Z-score by centering it at its country-specific mean and dividing by its country-specific standard deviation. Z-scores make it possible to interpret effect sizes as the impact of a 1 SD change in a covariate on the decadal change in life expectancy for countries in each starting LEB stratum.

The equation of the fixed effects model for LEB decade gain is:

1 $$ \mathrm{E}\;{\left[\Delta\;\mathrm{LEB}\ \mathrm{gains}\right]}_{\mathrm{i}\mathrm{t}}={\upalpha}_{\mathrm{i}}+{\upbeta}_1{\mathrm{LEB}}_{\mathrm{t}}+{\upbeta}_2{\mathrm{X}}_1{,}_{\mathrm{i}\mathrm{t}}+\dots +{\upbeta}_{\mathrm{k}}{\mathrm{X}}_{\mathrm{k}}{,}_{\mathrm{i}\mathrm{t}}+{\updelta}_1{\mathrm{D}}_1+{\updelta}_2{\mathrm{D}}_2+{\updelta}_3{\mathrm{D}}_3+{\updelta}_4{\mathrm{D}}_4+{\updelta}_5{\mathrm{D}}_5+{\upvarepsilon}_{\mathrm{i}\mathrm{t}} $$

Where α_i_ represents the unknown intercept for each country indexed by “i”; X_k,it_ represents a vector of k independent variables (k = 6 factors), for i countries (*i* = 139 countries) in t times (*t* = 59 years); _t_D_t_ represents binary time regressors for 5 decadal time-periods, with 1950–1959 excluded; and ε_it_ is the error term. Because each analysis is stratified to only include country-years of similar starting life expectancies, we would expect the benefits of accumulated biomedical technology over time to make the δ terms get progressively larger such that δ_5_>δ_4_>δ_3_>δ_2_>δ_1_.

The main model includes 139 countries, because HIV prevalence data were missing for 34 countries. An additional model was specified omitting HIV prevalence, to ensure the results are not an artifact of a selective subsample, results are on Table A-2-9 of the Additional file 1. This model includes 173 countries, the lists of countries included in both analysis are provided on Table A-2-1 and A-2-2 of the Additional file 1.

## Results

**Fig. 1:**
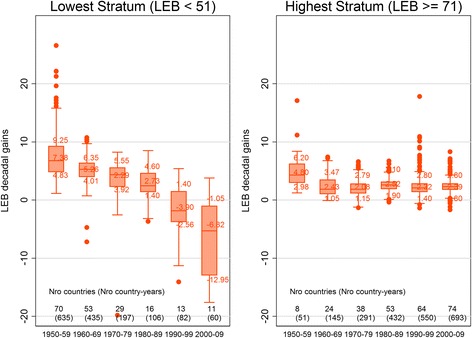
LEB decadal gains by decade, Lowest Stratum (LEB < 51) and Highest Stratum (LEB ≥ 71)

When we compare countries at a similar starting point of LEB, we find that the pace of growth of LEB has slowed over the last 60 years for countries at all levels of starting LEB. Countries with the lowest LEB (LEB < 51) had the greatest slowdown. Unadjusted results show that between the 1950s–2000s LEB decadal gains got smaller by approximately 14 years, from an average decade gain of 7.38 (SD 3.59) years in 1950–59 to an average decade loss of 6.82 (SD 6.64) years in 2000–09 (Fig. 1). For countries in the middle strata see Figure A-3-1 of the Additional file 1.

Regression-adjusted analysis also reconfirms that LEB decadal gains became progressively smaller over time (Table 2). This effect is consistent for countries at all starting life expectancies, but the effect size is greater for countries with lower life expectancies, for which the model explains 55.1% of the variation. In the lowest group, LEB decadal improvements in the 1960–69 decade were 4.171 (SE 0.473) years lower than in the 1950–59 decade (*P* < 0.001), and in the 2000–09 decade were 8.491 (SE 0.982) years lower than in the 1950–59 decade (*P* < 0.001). The deceleration of LEB growth among the group with the highest starting LEB was smaller, at 2.961 (SE 0.181) years gained per decade slower for the 1960s (P < 0.001) and 1.893 (SE 0.264) years gained per decade slower for the 2000s (P < 0.001), both compared to 1950s.

**Table 2:** The effects of LEB, income per capita, fertility, population density, CO2 emissions, HIV prevalence, and time on LEB decadal gains, 1950–2009, Fixed effects model

	LEB decade gains				
	Lowest Stratum (LEB < 51)	Stratum II (51 ≤ LEB < 61)	Stratum III (61 ≤ LEB < 71)	Highest Stratum (LEB ≥ 71)	Total
Decade 1960–69	−4.171*** (0.473)	−4.799*** (0.597)	−4.467*** (0.308)	−2.961*** (0.181)	−5.539*** (0.149)
Decade 1970–79	−6.385*** (0.597)	−7.567*** (0.644)	−5.657*** (0.377)	−1.982*** (0.183)	−7.449*** (0.189)
Decade 1980–89	−8.062*** (0.718)	− 10.54*** (0.687)	− 6.439*** (0.450)	− 1.693*** (0.194)	−9.055*** (0.238)
Decade 1990–99	−11.79*** (0.788)	− 12.31*** (0.735)	−7.633*** (0.504)	− 1.918*** (0.220)	− 10.34*** (0.292)
Decade 2000–09	− 8.491*** (0.982)	− 11.42*** (0.803)	− 7.283*** (0.585)	−1.893*** (0.264)	− 10.01*** (0.352)
Life Expectancy at Birth	2.004*** (0.132)	1.559*** (0.0812)	0.710*** (0.0903)	0.608*** (0.0693)	3.529*** (0.0753)
GDP per capita, 2005, PPP $	0.496*** (0.0940)	0.129** (0.0634)	0.130* (0.0788)	−0.0311 (0.0587)	−0.0520 (0.0439)
Population density per km^2^	−0.502** (0.195)	− 0.553*** (0.183)	− 0.882*** (0.0935)	−0.584*** (0.0492)	−1.496*** (0.100)
CO2 in tons per capita	0.0341 (0.110)	−0.0484 (0.0633)	−0.00566 (0.0706)	− 0.169*** (0.0295)	−0.175*** (0.0415)
Total Fertility Rate	−0.0836 (0.107)	−0.0781 (0.110)	0.107 (0.0893)	0.287*** (0.0372)	−0.0918* (0.0548)
HIV Prevalence	−0.355** (0.147)	−0.484*** (0.0910)	− 0.00773 (0.0747)	−0.0821** (0.0400)	− 0.427*** (0.0612)
Constant	8.956*** (0.543)	12.95*** (0.673)	9.408*** (0.404)	4.245*** (0.204)	10.93*** (0.233)
Observations	685	1460	1597	1908	6533
R-squared	0.551	0.552	0.337	0.321	0.478
Nro. of countries	34	63	81	63	139
Country Fixed Effects	Yes	Yes	Yes	Yes	Yes

Standard errors in parentheses

***p < 0.01, **p < 0.05, *p < 0.1

The effects of the factors used to explain improvement in LEB over time are shown in Table 2. As expected, income per capita had a positive effect. However, it is only statistically significant for countries with lower starting life expectancies, (*P* < 0.05). A 1 SD increase in GDP per capita is associated with a 0.496 (SE 0.0940) and 0.129 (SE 0.0634) increase in LEB decadal gains in countries with LEB below 51 and countries with LEB between 51 and 61, respectively.

CO2 emissions had a significant negative effect among countries with greatest longevity. An increase of 1 SD (roughly eight tons) in per capita CO2 emissions reduces LEB decadal gains by 0.169 (SE 0.0295) years. Fertility had a positive statistically significant effect for countries of the highest stratum, (*P* < 0.01). An increase of 1 SD (roughly 0.8) of TFR is associated with an increase of 0.287 (SE 0.0372) years in LEB decadal gains.

The HIV epidemic reduced LEB decadal gains in all countries, except those from stratum III. For example, a 1 SD increase in HIV prevalence had a reduction of 0.355 (SE 0.1470) years and 0.082 (SE 0.0400) years in LEB decadal gains among countries of the lowest stratum (*P* < 0.05) and highest stratum (P < 0.05), respectively.

When increasing the number of countries to 173 by omitting HIV prevalence as a control variable, a similar pattern of decelerating progress in LEB improvement was observed across all LEB strata, see Table A-2-9 of the Additional file 1.

## Discussion

Even though life expectancy increased year-by-year over the last 60 years, the gains became progressively smaller regardless of whether countries are starting with LEB below 51 or above 71 or any value in between. The reason for poor performance by the 11 countries with LEB below 51 in the 2000s cannot be because they were close to a longevity ceiling. The biological limits of the human life span cannot be a factor when life expectancy at birth is less than 51.

There are some important limitations. Many well known social determinants of health are not available for most countries back to 1950 and analyzing mortality at the level of a whole country has the disadvantage of not allowing any controls for individual level risk factors and experiences. But national level factors do affect national level mortality and are the reason population health indicators like LEB exist. Furthermore, aggregation of mortality to the level of a whole country allows fixed effects models to control for the time-invariant characteristics of a country and permits one to observe macroeconomic forces that affect the health of whole populations.

Our analysis spanned a period that included multiple scientific breakthroughs spreading new antibiotics, vaccines, and surgeries, as well as better treatments for AIDS, malaria, and TB, around the world. This period also included a substantial expansion of health care systems of nearly every country bringing dispensaries, clinics, and hospitals, as well as financial protection mechanisms. Thus, it is surprising that the analysis showed no evidence that time trends in LEB decadal gains improved over time regardless of starting life expectancy.

We can think of four potential explanations for this paradox. First, it is possible that pre-1970 data on life expectancy from HMD and WPP embody systematic assumptions differing from post-1970 data from IHME. Second, despite the inclusion of statistical controls for HIV prevalence, the HIV/AIDS era may have brought harmful spillover effects putting a brake on health improvements. Third, it is possible that the set of low life expectancy countries in 1950 differs systematically from the set of low life expectancy countries in 2000. Fourth, it is possible that the 1950s embodied an approach to population health different from the approaches used more recently.

### Data

It is possible to be misled by appending demographic data from different sources. Life expectancy estimates are inherently based on models, and the underlying age-specific mortality rates can be extensively modeled by demographers who are attempting to achieve best estimates consistent with all the evidence at hand. If the pre-1970 data came from models that overestimated life expectancy relative to the post-1970 source, our analysis should have found a step-off point only at 1970.

To assure that declining life expectancy is not simply the result of appending multiple databases, we repeated the analysis for 1970 onwards, using only IHME as source and continued to find statistically significant evidence of a slowdown in the pace of LEB gains, see Table A-2-8 of the Additional file 1. Thus, the effects are not solely due to the merging of life expectancy data from multiple sources.

### HIV/aids

It is possible that spillover effects from HIV impeded progress in improving health, even though our model controlled for HIV prevalence. It is also possible that HIV effects spill over to other decades and could have lagged effects for years into the future. This explanation is most relevant from 1990 onwards, and among countries from the lowest strata of starting LEB. From the 1980’s onward there was a slight improvement in HIV prevalence only among countries with a life expectancy between 61 and 71, decreasing from 0.86 (SD 2.45) in the 1980’s to 0.78 (SD 1.99) in the 2000’s. Countries with lowest LEB (LEB < 51) were the most affected by HIV, increasing their prevalence from 1.88 (SD 2.39) in the 1980’s to 17.03 (SD 7.83) in the 2000’s.

However, our analysis showed slowdowns in places and periods where HIV/AIDS was not prevalent enough to be a plausible explanation of LEB slowing pace. Additional file 1 Table A-2-10 confirms LEB growth deceleration since 1950 across regions where HIV prevalence was lower than Africa’s. We see evidence of decelerating rates of LEB growth in East Asia and Pacific, Europe and Central Asia, Latin America and the Caribbean, and Middle East and North Africa. Furthermore, LEB growth deceleration was occurring before and during the 1980s prior to the start of the HIV/AIDS era. Hence HIV may have contributed to slowing progress in LEB growth, but it cannot be the sole cause.

### Changed mix of countries

Stratification controls for LEB level in each country, and the fixed effects model can control for idiosyncratic features of every single country over time. However, our methods cannot adjust for unobservable features characterizing the shifting composition of countries within each grouping over time. For example, in the lowest group there are 635 country-years of observations on 70 countries (like Mali, Burkina Faso, and India) in the 1950s, and 60 country-years of observations on 11 countries (like Zimbabwe, Swaziland, and Lesotho) in the 2000s. It is plausible that the former larger set of countries in the 1950–59 is mainly composed of countries striving for better health, whereas the latter smaller set of countries that remain in the lowest group in 2000–09 include a higher proportion of fragile-states [22]. Fragile states have unique challenges to their health care systems, not only because of an increased burden of disease, but also because they have fragile governance and political conflicts, along with scarcity of health care workforce, and financial limitations.

While the shifting composition hypothesis is a compelling explanation for the declines in decadal life expectancy gains for the lowest stratum, it is somewhat less compelling for the highest stratum of life expectancy. Whereas the set of exceptional 12 countries achieving life expectancy above 70 by 1950–1959 would be enriched by high performers, all the 79 countries registering life expectancy above 70 in 2000–2009 would also have had to learn to strive to achieve higher life expectancies.

Explaining a declining pace of life expectancy gains around the world as reflecting a decline in the eagerness of societies and governments to improve population health should provoke critical reflection. Societies have shown great enthusiasm for signing declarations like the Millennium Development Goals and Sustainable Development Goals. Countries around the world have also shown a manifest willingness to increase governmental expenditure on delivering health personal services. They have had access to the very best policy advice on how to expand universal health service coverage to guarantee access to medical care for vulnerable groups. If the declining pace of progress indeed reflects a decline in political will among the least healthy countries, then the policy community is remiss in failing to harness any of the existing enthusiasm for higher government health spending into investments that will maintain historical rates of progress in the growth of life expectancy.

### Changed approach to population health

The final explanation is admittedly speculative, and would hold there has been a fundamental shift since the mid-twentieth century in the basic approach to improving population health in every country.

Infectious disease mortality reduced dramatically in high-income countries between 1880 and 1950, achieved as a result of what some call the “golden age of public health” [23, 24]. Improvements in housing, diet, the safety and quality of water, air, and food mediated by public health proponents were fundamental to these early twentieth century gains [25].

In the 1950s countries around the world began to have access to penicillin and other antibiotics. Following 1950, most countries prioritized efforts to scale-up health care services supply and life-saving medical treatments and vaccines. Following World War II, former colonies and less developed countries were challenged to simultaneously produce an effective public health workforce while meeting the mandate to introduce life-saving biomedical services.

In summary, a possible explanation for the slowdown in health progress is half a century of shifting emphasis away from covering populations with public health infrastructure to keep populations healthy and towards delivering clinical services to cure the sick. Unfortunately, the ideal evidence to test this conjecture would require data on each country’s expenditure on non-clinical public health related activities, and very few countries have this data [26]. The recent outbreaks of Ebola and Zika virus, and recurrent calls to strengthen public health, reflect a growing recognition of the unfinished agenda in building public health infrastructure around the world [27, 28].

## Conclusions

The pace of gains in life expectancy has slowed around the world since 1950, regardless of a country’s starting life expectancy level. The slowdown preceded the HIV/AIDS era and extends to regions that have not had high HIV/AIDS prevalence. Because government health spending has grown around the world, the slowdown cannot be due to a slowdown in health spending. Hence, restoring historical levels of LEB decadal growth is unlikely to be achieved by further increasing health spending along its current patterns. The slowdown coincided with an unprecedented growth in the discovery of life-saving biomedical technologies, so reversing the slowdown is unlikely to occur through expanded biomedical research. There are ample opportunities to restore a policy emphasis on public health approaches that worked well 60-years ago, when the world was poorer and sicker.

## Additional file

Additional file 1 **Table A-1-1.** LEB sources, by country and year. **Table A-2-1.** List of countries included in the analysis, when HIV is not included as a control variable. **Table A-2-2.** List of countries included in the analysis, when HIV is included as a control variable. **Table A-2-3.** List of countries by strata using a representative year for each decade, during the analysis countries were re-stratified to each LEB stratum every single year. **Table A-2-4.** Summary statistics, Lowest Stratum (LEB < 51). **Table A-2-5.** Summary statistics, Stratum II (51 ≤ LEB < 61). **Table A-2-6.** Summary statistics, Stratum III (61 ≤ LEB < 71). **Table A-2-7.** Summary statistics, Highest Stratum (LEB ≥ 71). **Table A-2-8.** F-tests of equality between decadal dummies parameters, 1960–69 against 2000–09 and 1980–89 against 2000–09. **Table A-2-9.** The effects of LEB, income per capita, fertility, population density, CO2 emissions, and time on LEB decadal gains, 1950–2009, Fixed effects model. **Table A-2-10.** LEB decade gains, by region, decade and LEB strata. **Table A-4-1.** Countries with HIV imputed values, FE regression (imputation A) and constant prevalence (imputation B). **Figure A-3-1.** LEB decadal gains by decade, Stratum II (51 ≤ LEB < 61) and Stratum III (61 ≤ LEB < 71). **Figure A-3-2.** LEB decadal gains distribution by strata, comparison between decades (PDF 1890 kb)

## Abbreviations

AIDS: Acquired immunodeficiency syndrome
CDIAC: Carbon dioxide information analysis center
CO2: Carbon dioxide
GDP: Gross domestic product
HIV: Human immunodeficiency virus
HMD: Human mortality database
IHME: Institute for health metrics and evaluation
IQR: Inter-quartile range
LEB: Life expectancy at birth
SE: Standard error
TFR: Total fertility rate
UN: United Nations
UNAIDS: Joint united nations programme on HIV and AIDS
WPP: World population prospects
