# Supplementary material for: The slowing pace of life expectancy gains since 1950
Source: BMC Public Health. 2018 Jan 17;18:151. doi: 10.1186/s12889-018-5058-9 (PMC5773026; doi:10.1186/s12889-018-5058-9)
Supplement: Additional file 1 — Table A-1-1. LEB sources, by country and year. Table A-2-1. List of countries included in the analysis, when HIV is not included as a control variable. Table A-2-2. List of countries included in the analysis, when HIV is included as a control variable. Table A-2-3. List of countries by strata using a representative year for each decade, during the analysis countries were re-stratified to each LEB stratum every single year. Table A-2-4. Summary statistics, Lowest Stratum (LEB < 51). Table A-2-5. Summary statistics, Stratum II (51 ≤ LEB < 61). Table A-2-6. Summary statistics, Stratum III (61 ≤ LEB < 71). Table A-2-7. Summary statistics, Highest Stratum (LEB ≥ 71). Table A-2-8. F-tests of equality between decadal dummies parameters, 1960–69 against 2000–09 and 1980–89 against 2000–09. Table A-2-9. The effects of LEB, income per capita, fertility, population density, CO2 emissions, and time on LEB decadal gains, 1950–2009, Fixed effects model. Table A-2-10. LEB decade gains, by region, decade and LEB strata. Table A-4-1. Countries with HIV imputed values, FE regression (imputation A) and constant prevalence (imputation B). Figure A-3-1. LEB decadal gains by decade, Stratum II (51 ≤ LEB < 61) and Stratum III (61 ≤ LEB < 71). Figure A-3-2. LEB decadal gains distribution by strata, comparison between decades (PDF 1890 kb) [file 12889_2018_5058_MOESM1_ESM.pdf]

Appendix

Content

1. Data Sources **1**

Life Expectancy at Birth 7

Real GDP per capita PPP 2005 7

Children per woman (total fertility) 7

Population density (per square kilometer) 8

Estimated HIV Prevalence % - (Ages 15-49) 9

2. Tables **10**

3. Figures **30**

4. HIV imputation **33**

Tables

Table A-1-1: LEB sources, by country and year 1

Table A-2-1: List of countries included in the analysis, when HIV is not included as a control variable 10

Table A-2-2: List of countries included in the analysis, when HIV is included as a control variable 12

Table A-2-3: List of countries by strata using a representative year for each decade, during the analysis countries were re-stratified to each LEB stratum every single year 14

Table A-2-4: Summary statistics, Lowest Stratum (LEB < 51) 19

Table A-2-5: Summary statistics, Stratum II (51 ≤ LEB < 61) 20

Table A-2-6: Summary statistics, Stratum III (61 ≤ LEB < 71) 21

Table A-2-7: Summary statistics, Highest Stratum (LEB ≥ 71) 22

Table A-2-8: F-tests of equality between decadal dummies parameters, 1960-69 against 2000-09 and 1980-89 against 2000-09 23

Table A-2-9: The effects of LEB, income per capita, fertility, population density, CO2 emissions, and time on LEB decadal gains, 1950 – 2009, Fixed effects model 24

Table A-2-10: LEB decade gains, by region, decade and LEB strata 25

Table A-4-1: Countries with HIV imputed values, FE regression (imputation A) and constant prevalence (imputation B) 33

Figures

Figure A-3-1: LEB decadal gains by decade, Stratum II (51≤LEB<61) and Stratum III (61≤LEB<71) 30

Figure A-3-2: LEB decadal gains distribution by strata, comparison between decades 31

1. Data Sources

Table A-1-1: LEB sources, by country and year

Country	IHME (2014)	WPP (2012)	HMD (2009)	WPP (2008)	Riley file	HMD (2013)	HMD (2011)	Interpolated	Estimate based on age-specific mortality	Gapminder guesstimate	Vallin et al. (2002)
Afghanistan	1970-2009	1950-1969									
Albania	1970-2009	1950-1969									
Algeria	1970-2009	1950-1969									
Angola	1970-2009	1950-1969									
Antigua and Barbuda	1970-2009	1950-1969									
Argentina	1970-2009	1950-1969									
Armenia	1970-2009	1950-1969									
Australia	1970-2009		1950-1969								
Austria	1970-2009		1950-1969								
Azerbaijan	1970-2009	1950-1969									
Bahamas	1970-2009	1950-1969									
Bahrain	1970-2009	1950-1969									
Bangladesh	1970-2009	1950-1969									
Barbados	1970-2009	1950-1969									
Belarus	1970-2009		1959-1969	1950-1958							
Belgium	1970-2009		1950-1969								
Belize	1970-2009	1950-1969									
Benin	1970-2009	1950-1969									
Bhutan	1970-2009	1950-1969									
Bolivia	1970-2009	1950-1969									
Bosnia and Herzegovina	1970-2009	1950-1969									
Botswana	1970-2009	1950-1969									
Brazil	1970-2009	1950-1969									
Brunei	1970-2009	1950-1969									
Bulgaria	1970-2009		1950-1969								
Burkina Faso	1970-2009	1950-1969									
Burundi	1970-2009	1950-1969									
Cambodia	1970-2009	1950-1969									

Country	IHME (2014)	WPP (2012)	HMD (2009)	WPP (2008)	Riley file	HMD (2013)	HMD (2011)	Interpolated	Estimate based on age-specific mortality	Gapminder guesstimate	Vallin et al. (2002)
Cameroon	1970-2009	1950-1969									
Canada	1970-2009		1950-1969								
Cape Verde	1970-2009	1950-1969									
Chad	1970-2009	1950-1969									
Chile	1970-2009			1950-1969							
China	1970-2009				1953-1969 (1)			1950-1952 (1)			
Colombia	1970-2009	1950-1969									
Comoros	1970-2009	1950-1969									
Congo, Dem. Rep.	1970-2009	1950-1969									
Congo, Rep.	1970-2009	1950-1969									
Costa Rica	1970-2009	1950-1969									
Cote d'Ivoire	1970-2009	1950-1969									
Croatia	1970-2009	1950-1969									
Cuba	1970-2009	1950-1969									
Cyprus	1970-2009	1950-1969									
Denmark	1970-2009		1950-1969								
Djibouti	1970-2009	1950-1969									
Ecuador	1970-2009	1950-1969									
Egypt	1970-2009	1950-1969									
El Salvador	1970-2009	1950-1969									
Equatorial Guinea	1970-2009	1950-1969									
Eritrea	1970-2009	1950-1969									
Estonia	1970-2009		1959-1969							1950-1958 (2)	
Ethiopia	1970-2009	1950-1956<br>1959-1963<br>1967-1969							1957-1958<br>1964-1966 (3)		
Fiji	1970-2009	1950-1969									
Finland	1970-2009		1950-1969								
France	1970-2009		1950-1969								
Gabon	1970-2009	1950-1969									
Gambia	1970-2009	1950-1969									
Georgia	1970-2009	1950-1969									

Country	IHME (2014)	WPP (2012)	HMD (2009)	WPP (2008)	Riley file	HMD (2013)	HMD (2011)	Interpolated	Estimate based on age-specific mortality	Gapminder guesstimate	Vallin et al. (2002)
Germany	1970-2009	1950-1955				1956-1969					
Ghana	1970-2009	1950-1969									
Greece	1970-2009	1950-1969									
Grenada	1970-2009	1950-1969									
Guatemala	1970-2009	1950-1969									
Guinea	1970-2009	1950-1969									
Guinea-Bissau	1970-2009	1960-1969						1950-1959 (4)			
Guyana	1970-2009	1950-1969									
Haiti	1970-2009	1950-1969									
Honduras	1970-2009	1950-1969									
Hong Kong, China	1970-2009	1950-1969									
Hungary	1970-2009		1950-1969								
Iceland	1970-2009		1950-1969								
India	1970-2009	1950-1969									
Indonesia	1970-2009	1950-1964<br>1967-1969							1965-1966 (5)		
Iran	1970-2009	1950-1969									
Iraq	1970-2009	1950-1969									
Ireland	1970-2009		1950-1969								
Israel	1970-2009			1950-1969							
Italy	1970-2009		1950-1969								
Jamaica	1970-2009	1950-1969									
Japan	1970-2009		1950-1969								
Jordan	1970-2009	1950-1969									
Kazakhstan	1970-2009	1950-1969									
Kenya	1970-2009	1950-1969									
Kiribati	1970-2009	1950-1969									
Kuwait	1970-2009	1950-1969									
Latvia	1970-2009	1950-1958				1959-1969					
Lebanon	1970-2009	1950-1969									
Lesotho	1970-2009	1950-1969									
Liberia	1970-2009	1950-1969									

Country	IHME (2014)	WPP (2012)	HMD (2009)	WPP (2008)	Riley file	HMD (2013)	HMD (2011)	Interpolated	Estimate based on age-specific mortality	Gapminder guesstimate	Vallin et al. (2002)
Libya	1970-2009	1950-1969									
Lithuania	1970-2009		1959-1969	1950-1958							
Luxembourg	1970-2009		1960-1969	1950-1959							
Macao, China	1970-2009	1950-1969									
Macedonia, FYR	1970-2009	1950-1969									
Madagascar	1970-2009	1950-1969									
Malawi	1970-2009	1950-1969									
Malaysia	1970-2009	1950-1969									
Maldives	1970-2009	1950-1969									
Mali	1970-2009	1950-1969									
Malta	1970-2009	1950-1969									
Mauritania	1970-2009	1950-1969									
Mauritius	1970-2009	1950-1969									
Mexico	1970-2009	1950-1969									
Micronesia, Fed. Sts.	1970-2009	1950-1969									
Moldova	1970-2009	1950-1969									
Mongolia	1970-2009	1950-1969									
Montenegro	1970-2009	1950-1969									
Morocco	1970-2009	1950-1969									
Mozambique	1970-2009	1950-1969									
Namibia	1970-2009	1950-1969									
Nepal	1970-2009	1950-1969									
Netherlands	1970-2009		1950-1969								
New Zealand	1970-2009		1950-1969								
Nicaragua	1970-2009	1950-1969									
Niger	1970-2009	1950-1969									
Nigeria	1970-2009	1950-1966							1967-1969 (6)		
Norway	1970-2009		1950-1969								
Oman	1970-2009	1950-1969									
Pakistan	1970-2009	1950-1969									
Panama	1970-2009	1950-1969									
Papua New Guinea	1970-2009	1950-1969									

Country	IHME (2014)	WPP (2012)	HMD (2009)	WPP (2008)	Riley file	HMD (2013)	HMD (2011)	Interpolated	Estimate based on age-specific mortality	Gapminder guesstimate	Vallin et al. (2002)
Paraguay	1970-2009	1950-1969									
Peru	1970-2009	1950-1969									
Philippines	1970-2009	1950-1969									
Poland	1970-2009		1958-1969	1950-1957							
Portugal	1970-2009		1950-1969								
Qatar	1970-2009	1950-1969									
Romania	1970-2009	1950-1969									
Russia	1970-2009		1959-1969		1950-1958 (7)						
Rwanda	1970-2009			1950-1969							
Samoa	1970-2009	1950-1969									
Sao Tome and Principe	1970-2009	1950-1969									
Saudi Arabia	1970-2009	1950-1969									
Senegal	1970-2009	1950-1969									
Serbia	1970-2009	1950-1969									
Seychelles	1970-2009	1950-1969									
Sierra Leone	1970-2009	1950-1969									
Singapore	1970-2009	1950-1969									
Slovak Republic	1970-2009		1950-1969								
Slovenia	1970-2009			1950-1969							
Solomon Islands	1970-2009	1950-1969									
Somalia	1970-2009	1950-1969									
South Africa	1970-2009	1950-1969									
Spain	1970-2009		1950-1969								
Sri Lanka	1970-2009	1950-1969									
Sudan	1970-2009	1950-1969									
Suriname	1970-2009	1950-1969									
Swaziland	1970-2009	1950-1969									
Sweden	1970-2009		1950-1969								
Switzerland	1970-2009		1950-1969								
Syria	1970-2009	1950-1969									
Tajikistan	1970-2009	1950-1969									
Tanzania	1970-2009	1950-1969									

Country	IHME (2014)	WPP (2012)	HMD (2009)	WPP (2008)	Riley file	HMD (2013)	HMD (2011)	Interpolated	Estimate based on age-specific mortality	Gapminder guesstimate	Vallin et al. (2002)
Thailand	1970-2009	1950-1969									
Timor-Leste	1970-2009	1950-1969									
Togo	1970-2009	1950-1969									
Tonga	1970-2009	1950-1969									
Trinidad and Tobago	1970-2009	1950-1969									
Tunisia	1970-2009	1950-1969									
Turkey	1970-2009	1950-1969									
Turkmenistan	1970-2009	1950-1969									
Uganda	1970-2009	1950-1969									
Ukraine	1970-2009		1959-1969								1950-1958
United Arab Emirates	1970-2009	1950-1969									
United Kingdom	1970-2009		1950-1969								
United States	1970-2009						1950-1969				
Uruguay	1970-2009	1950-1969									
Uzbekistan	1970-2009	1950-1969									
Vanuatu	1970-2009	1950-1969									
Venezuela	1970-2009	1950-1969									
Vietnam	1970-2009	1950-1969									
Zambia	1970-2009	1950-1969									
Zimbabwe	1970-2009	1950-1969									

Source: Gapminder Documentation 004 (GD004) - version 8 (2016)

Life Expectancy at Birth

According to Gapminder Documentation 004 (GD004) - version 8 [1], f or 173 countries used in the analysis, LEB sources were (sorted by number of country-year observations):

1. IHME [2] (2014), 6,920 country-year observations. "Institute for Health Metrics and Evaluation – University of Washington." Retrieved file IHME_GBD_2013_LIFE_EXPECTANCY_1970_2013_Y2014M12D17 from https://cloud.ihme.washington.edu/index.php/s/b89390325f728bbd99de0356d3be6900.
2. WPP [3] (2012), 2,715 country-year observations. "United Nations Population Division. World Population Prospects: The 2012 Revision. Life expectancy at birth, both sexes."
3. HMD [4] (2009), 517 country-year observations. "The Human Mortality Database. Joint project of the Department of Demography at the University of California, Berkeley, USA, and at the Max Planck Institute for Demographic Research in Rostock. Downloaded in 2009."
4. WPP [5] (2008), 116 country-year observations. "United Nations Population Division. World Population Prospects: The 2006 Revision. Life expectancy at birth, both sexes."
5. Riley file, 26 country-year observations. "Estimates from in total over 700 different sources, compiled by Professor James Riley, and described in www.lifetable.de/RileyBib.htm". Table A-1 shows a detail explanation of the estimate (Notes).
6. HMD [6] (2013), 25 country-year observations. "The Human Mortality Database. Joint project of the Department of Demography at the University of California, Berkeley, USA, and at the Max Planck Institute for Demographic Research in Rostock. Downloaded in 2013, July. There have been some additions and some minor revisions since 2011."
7. HMD [7] (2011), 20 country-year observations. "The Human Mortality Database. Joint project of the Department of Demography at the University of California, Berkeley, USA, and at the Max Planck Institute for Demographic Research in Rostock. Downloaded in 2011. There has been some additions and some minor revisions since 2009."
8. Interpolated, 13 country-year observations. Table A-1 shows a detail explanation of the estimate (Notes).
9. Gapminder guesstimate, 10 country-year observations. Table A-1 shows a detail explanation of the estimate (Notes).
10. Estimate based on age-specific mortality from Estonian Interuniversity Population Research Centre (database), 9 country-year observations. Table A-1 shows a detail explanation of the estimate (Notes).
11. Vallin et al. (2002), 9 country-year observations. "Vallin etal (2002), "A new estimate of Ukrainian population losses during the crises of the 1930s and 1940s". Population Studies: A Journal of Demography, Volume 56, Issue 3."

Real GDP per capita PPP 2005

According to Data in Gapminder World [8], information was retrieved from:

Alan Heston, Robert Summers and Bettina Aten [8], Penn World Table Version 7.1, Center for International Comparisons of Production, Income and Prices at the University of Pennsylvania, July 2012. Version: September, 2012

Children per woman (total fertility)

According to Gapminder Documentation 008 (GD008) - version 6 [9], Total Fertility Rate (TFR) main source of data is the UN fertility dataset, which covers the period between 1950 and 2008. This version, has the latest UN-population World Population Prospect, as of 2015 October.

For the 173 countries used in the analysis, TFR sources were (sorted by number of country-year observations):

1. WPP [10] (2013), 9,604 country-year observations (equivalent to 92.5% of total observations). "United Nations, Department of Economic and Social Affairs, Population Division (2013). World Population Prospects: The 2012 Revision, CD-ROM Edition. File INT/1: Interpolated demographic indicators by major area, region and country, annually for 1950-2100." Retrieved in August 2013.
2. HFD (2013), 177 country-year observations. "Human Fertility Database. Max Planck Institute for Demographic Research (Germany) and Vienna Institute of Demography (Austria)." Available at: http://www.humanfertility.org
3. HFD (2012), 60 country-year observations. "Human Fertility Database. Max Planck Institute for Demographic Research (Germany) and Vienna Institute of Demography (Austria)." Retrieved in September 2012. Available at: www.humanfertility.org
4. Statistics Denmark (2013), 60 country-year observations. "Statistics Denmark (2013). Somehwere on their homepage: "FOD3: Age-specific fertility rates, total fertility rates, gross and net reproduction rate"." Retrieved in August 2013. Available at: http://www.statbank.dk/statbank5a/default.asp?w=1280

5. Statistics Finland (2013), 60 country-year observations. "Statistics Finland (2013), on-line table "Total fertility rate 1776-2012"." Retrieved in August 2013.
6. ABS (2008), 57 country-year observations. "Australian Bureau of Statistics (2008). Somewhere on their homepage: "3105.0.65.001 Australian Historical Population Statistics, 2008. Table 5. Births"." Available at: http://www.abs.gov.au/
7. Converted from CBR, 55 country-year observations.
8. Statistics Netherlands (2009), 55 country-year observations. "statistics Netherlands (2009). Somewhere on homepage." Available at: http://statline.cbs.nl/StatWeb
9. HFD (2015), 51 country-year observations. "Human Fertility Database. Max Planck Institute for Demographic Research (Germany) and Vienna Institute of Demography (Austria)." Retrieved in January 2015. Available at: http://www.humanfertility.org
10. Statistics Norway (2013), 42 country-year observations. "Statistics Norway (2013). Somewhere on homepage: "Table: 04232: Total fertility rate, women (C)"."
    Retrieved in August 2013. Available at: https://www.ssb.no/statistikkbanken/
11. e2307, 33 country-year observations. "Coale & Li (1987), "Basic data on fertility in the provinces of China, 1940-82", Papers of the east-west population institute." Available at: http://scholarspace.manoa.hawaii.edu/bitstream/handle/10125/22799/PapersOfTheEastWestPopulationInstituteNO.104BasicDataOnFertilityInTheProvincesOfChina1940-821987[pdfa].PDF?sequence=1
12. Statistics Singapore, 29 country-year observations.
13. Chesnais, 18 country-year observations. "Chesnais, Jean-Claude. (1992) The Demographic Transition: Stages, Patterns, and Economic Implications. A Longitudinal Study of Sixty-Seven Countries Covering the Period 1720-1984 Jean-Claude Chesnais Oxford : Clarendon Press , 1992"
14. Statistics Armenia (2013), 10 country-year observations. "Statistics Canada (2013). Table102-4505 - Crude birth rate, age-specific and total fertility rates (live births), Canada, provinces and territories, annual (rate), CANSIM (database). (accessed: 2013-08-23)." Retrieved in August 2013. Available at: http://www5.statcan.gc.ca/cansim/a05?lang=eng&id=1024505&paSer=&pattern=102-4505&stByVal=1&csid=
15. Zakharov, 9 country-year observations. "Zakharov (2008); "Russian federation: from the first to second demographic transition"; Demographic research 19 (24)." Available at: http://www.demographic-research.org/volumes/vol19/24/
16. Interpolated, 6 country-year observations.
    Romania (1950-1955):
    GD008: "WPP indicate lower values in the 1950s and implies a sharp discontinuity 1949-50."
17. Statistics Armenia (2009), 5 country-year observations. "Statistics Canada (2009), somewhere on their homepage: "Section B: Vital Statistics and Health"." Available at: http://www.statcan.gc.ca/pub/11-516-x/pdf/5500093-eng.pdf
18. Statistics Netherlands (2013), 5 country-year observations. "Statistics Netherlands (2013). Somewhere on homepage." Retrieved in August 2013. Available at: http://statline.cbs.nl
19. ABS (2013), 3 country-year observations. "Australian Bureau of Statistics (2013), Somewhere on homepage: "3301.0 - Births, Australia, 2011. Table 11.1 Age-specific fertility rates and total fertility rate, Single year of age of mother–Australia–1975 to 2011"." Retrieved in August 2013. Available at: http://www.abs.gov.au/AUSSTATS/abs@.nsf/DetailsPage/3301.02011?OpenDocument
20. Statistics Canada (2013), 2 country-year observations. "Statistics Canada (2013). Table102-4505 - Crude birth rate, age-specific and total fertility rates (live births), Canada, provinces and territories, annual (rate), CANSIM (database). (accessed: 2013-08-23)." Retrieved in August 2013. Available at: http://www5.statcan.gc.ca/cansim/a05?lang=eng&id=1024505&paSer=&pattern=102-4505&stByVal=1&csid.

Population density (per square kilometer)

According to Data in Gapminder World [11], information was retrieved from:

UN Population Division. World Population Prospects, the 2010 Revision. Population density (per square km). Version: August, 2013

Per capita CO2 emissions (metric tons of CO2)

According to Data in Gapminder World [12], information was retrieved from:

CDIAC (Carbon Dioxide Information Analysis Center). CDIAC (Carbon Dioxide Information Analysis Center) - nation.1751_2009.csv. http://cdiac.ornl.gov/trends/emis/meth_reg.html. Version: January, 2015.

Estimated HIV Prevalence % - (Ages 15-49)

According to Data in Gapminder World [13], information was retrieved from:

UNAIDS/WHO. UNAIDS online database, Estimated HIV Prevalence % - (Ages 15-49). Version: April, 2013.

For a detail description on HIV indicators look on Gapminder Documentation 006 (GD006) – version 1.

2. Tables

Table A-2-1: List of countries included in the analysis, when HIV is not included as a control variable

No.	Country	ISO Code	No.	Country	ISO Code
1	Afghanistan	AFG	88	Lebanon	LBN
2	Angola	AGO	89	Liberia	LBR
3	Albania	ALB	90	Libya	LBY
4	United Arab Emirates	ARE	91	Sri Lanka	LKA
5	Argentina	ARG	92	Lesotho	LSO
6	Armenia	ARM	93	Lithuania	LTU
7	Antigua and Barbuda	ATG	94	Luxembourg	LUX
8	Australia	AUS	95	Latvia	LVA
9	Austria	AUT	96	Macao, China	MAC
10	Azerbaijan	AZE	97	Morocco	MAR
11	Burundi	BDI	98	Moldova	MDA
12	Belgium	BEL	99	Madagascar	MDG
13	Benin	BEN	100	Maldives	MDV
14	Burkina Faso	BFA	101	Mexico	MEX
15	Bangladesh	BGD	102	Macedonia, FYR	MKD
16	Bulgaria	BGR	103	Mali	MLI
17	Bahrain	BHR	104	Malta	MLT
18	Bahamas	BHS	105	Montenegro	MNE
19	Bosnia and Herzegovina	BIH	106	Mongolia	MNG
20	Belarus	BLR	107	Mozambique	MOZ
21	Belize	BLZ	108	Mauritania	MRT
22	Bolivia	BOL	109	Mauritius	MUS
23	Brazil	BRA	110	Malawi	MWI
24	Barbados	BRB	111	Malaysia	MYS
25	Brunei	BRN	112	Namibia	NAM
26	Bhutan	BTN	113	Niger	NER
27	Botswana	BWA	114	Nigeria	NGA
28	Canada	CAN	115	Nicaragua	NIC
29	Switzerland	CHE	116	Netherlands	NLD
30	Chile	CHL	117	Norway	NOR
31	China	CHN	118	Nepal	NPL
32	Cote d'Ivoire	CIV	119	New Zealand	NZL
33	Cameroon	CMR	120	Oman	OMN
34	Congo, Rep.	COG	121	Pakistan	PAK
35	Colombia	COL	122	Panama	PAN
36	Comoros	COM	123	Peru	PER
37	Cape Verde	CPV	124	Philippines	PHL
38	Costa Rica	CRI	125	Papua New Guinea	PNG
39	Cuba	CUB	126	Poland	POL
40	Cyprus	CYP	127	Portugal	PRT
41	Germany	DEU	128	Paraguay	PRY
42	Djibouti	DJI	129	Qatar	QAT
43	Denmark	DNK	130	Romania	ROM
44	Algeria	DZA	131	Russia	RUS
45	Ecuador	ECU	132	Rwanda	RWA
46	Egypt	EGY	133	Saudi Arabia	SAU
47	Eritrea	ERI	134	Sudan	SDN

No.	Country	ISO Code	No.	Country	ISO Code
48	Spain	ESP	135	Senegal	SEN
49	Estonia	EST	136	Singapore	SGP
50	Ethiopia	ETH	137	Solomon Islands	SLB
51	Finland	FIN	138	Sierra Leone	SLE
52	Fiji	FJI	139	El Salvador	SLV
53	France	FRA	140	Somalia	SOM
54	Micronesia, Fed. Sts.	FSM	141	Serbia	SRB
55	Gabon	GAB	142	Sao Tome and Principe	STP
56	United Kingdom	GBR	143	Suriname	SUR
57	Georgia	GEO	144	Slovak Republic	SVK
58	Ghana	GHA	145	Slovenia	SVN
59	Guinea	GIN	146	Sweden	SWE
60	Gambia	GMB	147	Swaziland	SWZ
61	Guinea-Bissau	GNB	148	Seychelles	SYC
62	Equatorial Guinea	GNQ	149	Syria	SYR
63	Greece	GRC	150	Chad	TCD
64	Grenada	GRD	151	Togo	TGO
65	Guatemala	GTM	152	Thailand	THA
66	Guyana	GUY	153	Tajikistan	TJK
67	Hong Kong, China	HKG	154	Turkmenistan	TKM
68	Honduras	HND	155	Timor-Leste	TLS
69	Croatia	HRV	156	Tonga	TON
70	Haiti	HTI	157	Trinidad and Tobago	TTO
71	Hungary	HUN	158	Tunisia	TUN
72	Indonesia	IDN	159	Turkey	TUR
73	India	IND	160	Tanzania	TZA
74	Ireland	IRL	161	Uganda	UGA
75	Iran	IRN	162	Ukraine	UKR
76	Iraq	IRQ	163	Uruguay	URY
77	Iceland	ISL	164	United States	USA
78	Israel	ISR	165	Uzbekistan	UZB
79	Italy	ITA	166	Venezuela	VEN
80	Jamaica	JAM	167	Vietnam	VNM
81	Jordan	JOR	168	Vanuatu	VUT
82	Japan	JPN	169	Samoa	WSM
83	Kazakhstan	KAZ	170	South Africa	ZAF
84	Kenya	KEN	171	Congo, Dem. Rep.	ZAR
85	Cambodia	KHM	172	Zambia	ZMB
86	Kiribati	KIR	173	Zimbabwe	ZWE
87	Kuwait	KWT			

Table A-2-2: List of countries included in the analysis, when HIV is included as a control variable

No.	Country	ISO Code	No.	Country	ISO Code
1	Angola	AGO	71	Liberia	LBR
2	Argentina	ARG	72	Sri Lanka	LKA
3	Armenia	ARM	73	Lesotho	LSO
4	Australia	AUS	74	Lithuania	LTU
5	Austria	AUT	75	Luxembourg	LUX
6	Azerbaijan	AZE	76	Latvia	LVA
7	Burundi	BDI	77	Morocco	MAR
8	Belgium	BEL	78	Moldova	MDA
9	Benin	BEN	79	Madagascar	MDG
10	Burkina Faso	BFA	80	Maldives	MDV
11	Bangladesh	BGD	81	Mexico	MEX
12	Bulgaria	BGR	82	Mali	MLI
13	Bahamas	BHS	83	Malta	MLT
14	Belarus	BLR	84	Mongolia	MNG
15	Belize	BLZ	85	Mozambique	MOZ
16	Bolivia	BOL	86	Mauritania	MRT
17	Brazil	BRA	87	Mauritius	MUS
18	Barbados	BRB	88	Malawi	MWI
19	Bhutan	BTN	89	Malaysia	MYS
20	Botswana	BWA	90	Namibia	NAM
21	Canada	CAN	91	Niger	NER
22	Switzerland	CHE	92	Nigeria	NGA
23	Chile	CHL	93	Nicaragua	NIC
24	Cote d'Ivoire	CIV	94	Netherlands	NLD
25	Cameroon	CMR	95	Norway	NOR
26	Congo, Rep.	COG	96	Nepal	NPL
27	Colombia	COL	97	New Zealand	NZL
28	Comoros	COM	98	Oman	OMN
29	Costa Rica	CRI	99	Pakistan	PAK
30	Cuba	CUB	100	Panama	PAN
31	Germany	DEU	101	Peru	PER
32	Djibouti	DJI	102	Philippines	PHL
33	Denmark	DNK	103	Papua New Guinea	PNG
34	Algeria	DZA	104	Poland	POL
35	Ecuador	ECU	105	Portugal	PRT
36	Egypt	EGY	106	Paraguay	PRY
37	Eritrea	ERI	107	Qatar	QAT
38	Spain	ESP	108	Romania	ROM
39	Estonia	EST	109	Russia	RUS
40	Finland	FIN	110	Rwanda	RWA
41	Fiji	FJI	111	Sudan	SDN
42	France	FRA	112	Senegal	SEN
43	Gabon	GAB	113	Singapore	SGP
44	United Kingdom	GBR	114	Sierra Leone	SLE
45	Georgia	GEO	115	El Salvador	SLV
46	Ghana	GHA	116	Somalia	SOM
47	Guinea	GIN	117	Serbia	SRB
48	Gambia	GMB	118	Suriname	SUR
49	Guinea-Bissau	GNB	119	Slovak Republic	SVK
50	Equatorial Guinea	GNQ	120	Slovenia	SVN
51	Greece	GRC	121	Sweden	SWE
52	Guatemala	GTM	122	Swaziland	SWZ
53	Guyana	GUY	123	Chad	TCD
54	Honduras	HND	124	Togo	TGO

No.	Country	ISO Code	No.	Country	ISO Code
55	Croatia	HRV	125	Thailand	THA
56	Haiti	HTI	126	Tajikistan	TJK
57	Hungary	HUN	127	Trinidad and Tobago	TTO
58	Indonesia	IDN	128	Tunisia	TUN
59	India	IND	129	Turkey	TUR
60	Ireland	IRL	130	Tanzania	TZA
61	Iran	IRN	131	Uganda	UGA
62	Iceland	ISL	132	Ukraine	UKR
63	Israel	ISR	133	Uruguay	URY
64	Italy	ITA	134	United States	USA
65	Jamaica	JAM	135	Uzbekistan	UZB
66	Japan	JPN	136	Vietnam	VNM
67	Kazakhstan	KAZ	137	South Africa	ZAF
68	Kenya	KEN	138	Zambia	ZMB
69	Cambodia	KHM	139	Zimbabwe	ZWE
70	Lebanon	LBN			

Table A-2-3: List of countries by strata using a representative year for each decade, during the analysis countries were re-stratified to each LEB stratum every single year

	Year 1955	Year 1965	Year 1975	Year 1985	Year 1995	Year 2005
**Lowest Stratum (LEB < 51)**	Algeria	Angola	Angola	Angola	Angola	Guinea-Bissau
	Angola	Bangladesh	Benin	Burundi	Burundi	Lesotho
	Bangladesh	Benin	Bhutan	Eritrea	Equatorial Guinea	Malawi
	Benin	Bhutan	Burkina Faso	Guinea	Malawi	Swaziland
	Bhutan	Bolivia	Burundi	Guinea-Bissau	Mali	Zambia
	Bolivia	Burkina Faso	Cambodia	Malawi	Niger	Zimbabwe
	Burkina Faso	Burundi	Equatorial Guinea	Mali	Rwanda	
	Burundi	Cambodia	Eritrea	Mozambique	Uganda	
	Cambodia	Cameroon	Guinea	Niger	Zambia	
	Cameroon	Chad	Guinea-Bissau	Rwanda		
	Chad	Comoros	Haiti	Sierra Leone		
	Comoros	Congo, Rep.	India			
	Congo, Rep.	Cote d'Ivoire	Liberia			
	Cote d'Ivoire	Egypt	Malawi			
	Djibouti	Equatorial Guinea	Mali			
	Egypt	Eritrea	Mozambique			
	El Salvador	Gabon	Nepal			
	Equatorial Guinea	Gambia	Niger			
	Eritrea	Guatemala	Rwanda			
	Gabon	Guinea	Senegal			
	Gambia	Guinea-Bissau	Sierra Leone			
	Ghana	Haiti	Somalia			
	Guatemala	India				
	Guinea	Indonesia				
	Guinea-Bissau	Liberia				
	Haiti	Madagascar				
	Honduras	Malawi				
	India	Maldives				
	Indonesia	Mali				
	Iran	Mozambique				
	Kenya	Nepal				
	Lesotho	Niger				
	Liberia	Nigeria				
	Madagascar	Oman				
	Malawi	Pakistan				
	Maldives	Peru				
	Mali	Rwanda				
	Mauritania	Senegal				
	Mongolia	Sierra Leone				
	Morocco	Somalia				
	Mozambique	Togo				
	Namibia	Uganda				

	Year 1955	Year 1965	Year 1975	Year 1985	Year 1995	Year 2005
	Nepal					
	Nicaragua					
	Niger					
	Nigeria					
	Oman					
	Pakistan					
	Papua New Guinea					
	Peru					
	Rwanda					
	Senegal					
	Sierra Leone					
	Somalia					
	South Africa					
	Sudan					
	Swaziland					
	Tanzania					
	Togo					
	Tunisia					
	Turkey					
	Uganda					
	Zambia					
**Stratum II (51 ≤ LEB < 61)**	Armenia	Algeria	Algeria	Bangladesh	Benin	Angola
	Azerbaijan	Azerbaijan	Bangladesh	Benin	Burkina Faso	Botswana
	Bahamas	Botswana	Bolivia	Bhutan	Cambodia	Burkina Faso
	Belize	Brazil	Cameroon	Bolivia	Cameroon	Burundi
	Botswana	Chile	Chad	Burkina Faso	Chad	Cameroon
	Brazil	Djibouti	Comoros	Cambodia	Comoros	Chad
	Chile	Ecuador	Congo, Rep.	Cameroon	Congo, Rep.	Congo, Rep.
	Colombia	El Salvador	Cote d'Ivoire	Chad	Cote d'Ivoire	Cote d'Ivoire
	Ecuador	Ghana	Djibouti	Comoros	Djibouti	Djibouti
	Fiji	Honduras	Egypt	Congo, Rep.	Eritrea	Equatorial Guinea
	Georgia	Iran	Gabon	Cote d'Ivoire	Gabon	Eritrea
	Guyana	Kazakhstan	Gambia	Djibouti	Gambia	Gabon
	Jamaica	Kenya	Ghana	Equatorial Guinea	Ghana	Guinea
	Kazakhstan	Lesotho	Guatemala	Gabon	Guinea	Haiti
	Malaysia	Mauritania	Indonesia	Gambia	Guinea-Bissau	Kenya
	Mauritius	Mexico	Iran	Ghana	Haiti	Liberia
	Mexico	Mongolia	Kenya	Haiti	India	Mali
	Moldova	Morocco	Lesotho	India	Kenya	Mozambique
	Panama	Namibia	Madagascar	Lesotho	Lesotho	Namibia
	Philippines	Nicaragua	Maldives	Liberia	Liberia	Niger
	Qatar	Papua New Guinea	Mauritania	Madagascar	Madagascar	Nigeria
	Serbia	South Africa	Mongolia	Mauritania	Mongolia	Papua New Guinea
	Sri Lanka	Sudan	Morocco	Mongolia	Mozambique	Rwanda
	Suriname	Swaziland	Namibia	Nepal	Namibia	Sierra Leone

	Year 1955	Year 1965	Year 1975	Year 1985	Year 1995	Year 2005
	Tajikistan	Tajikistan	Nigeria	Nigeria	Nigeria	Somalia
	Thailand	Tanzania	Oman	Papua New Guinea	Papua New Guinea	South Africa
	Trinidad and Tobago	Tunisia	Pakistan	Senegal	Senegal	Tanzania
	Uzbekistan	Turkey	Papua New Guinea	Somalia	Sierra Leone	Togo
	Vietnam	Uzbekistan	South Africa	Sudan	Somalia	Uganda
	Zimbabwe	Vietnam	Sudan	Swaziland	Swaziland	
		Zambia	Swaziland	Tanzania	Tanzania	
		Zimbabwe	Tajikistan	Togo	Togo	
			Tanzania	Uganda	Zimbabwe	
			Togo	Zambia		
			Turkey			
			Uganda			
			Zambia			
			Zimbabwe			
**Stratum III (61 ≤ LEB < 71)**	Argentina	Argentina	Argentina	Algeria	Armenia	Azerbaijan
	Australia	Armenia	Armenia	Armenia	Azerbaijan	Bangladesh
	Austria	Austria	Azerbaijan	Azerbaijan	Bahamas	Belarus
	Barbados	Bahamas	Bahamas	Bahamas	Bangladesh	Belize
	Belarus	Barbados	Barbados	Botswana	Belarus	Benin
	Belgium	Belgium	Belize	Brazil	Bhutan	Bhutan
	Bulgaria	Belize	Botswana	Colombia	Bolivia	Bolivia
	Canada	Bulgaria	Brazil	Ecuador	Botswana	Cambodia
	Costa Rica	Colombia	Chile	Egypt	Brazil	Comoros
	Croatia	Costa Rica	Colombia	El Salvador	Bulgaria	Egypt
	Cuba	Croatia	Croatia	Estonia	Egypt	Fiji
	Estonia	Cuba	Ecuador	Fiji	Estonia	Gambia
	Finland	Fiji	El Salvador	Georgia	Fiji	Ghana
	France	Finland	Estonia	Guatemala	Guatemala	Guatemala
	Germany	Georgia	Fiji	Guyana	Guyana	Guyana
	Greece	Germany	Georgia	Honduras	Honduras	Honduras
	Hungary	Guyana	Guyana	Hungary	Hungary	India
	Ireland	Hungary	Honduras	Indonesia	Indonesia	Indonesia
	Israel	Ireland	Hungary	Iran	Iran	Kazakhstan
	Italy	Israel	Jamaica	Kazakhstan	Kazakhstan	Latvia
	Japan	Italy	Kazakhstan	Kenya	Latvia	Madagascar
	Latvia	Jamaica	Latvia	Latvia	Lithuania	Mauritania
	Lebanon	Japan	Lebanon	Lebanon	Maldives	Moldova
	Lithuania	Lebanon	Malaysia	Malaysia	Mauritania	Mongolia
	Luxembourg	Luxembourg	Mauritius	Maldives	Mauritius	Nepal
	Malta	Malaysia	Mexico	Mauritius	Moldova	Pakistan
	New Zealand	Malta	Moldova	Mexico	Morocco	Philippines
	Paraguay	Mauritius	Nicaragua	Moldova	Nepal	Russia
	Poland	Moldova	Paraguay	Morocco	Pakistan	Senegal
	Portugal	Panama	Peru	Namibia	Philippines	Sudan
	Romania	Paraguay	Philippines	Nicaragua	Romania	Suriname

	Year 1955	Year 1965	Year 1975	Year 1985	Year 1995	Year 2005
	Russia	Philippines	Poland	Oman	Russia	Tajikistan
	Singapore	Poland	Portugal	Pakistan	South Africa	Trinidad and Tobago
	Slovak Republic	Portugal	Qatar	Peru	Sudan	Ukraine
	Slovenia	Qatar	Romania	Philippines	Suriname	Uzbekistan
	Spain	Romania	Russia	Poland	Tajikistan	
	Switzerland	Russia	Serbia	Romania	Thailand	
	Ukraine	Serbia	Singapore	Russia	Trinidad and Tobago	
	United Kingdom	Singapore	Slovak Republic	Slovak Republic	Turkey	
	United States	Slovak Republic	Slovenia	South Africa	Ukraine	
	Uruguay	Slovenia	Sri Lanka	Sri Lanka	Uzbekistan	
		Spain	Suriname	Suriname	Vietnam	
		Sri Lanka	Thailand	Tajikistan		
		Suriname	Trinidad and Tobago	Thailand		
		Thailand	Tunisia	Trinidad and Tobago		
		Trinidad and Tobago	Ukraine	Tunisia		
		Ukraine	Uruguay	Turkey		
		United States	Uzbekistan	Ukraine		
		Uruguay	Vietnam	Uzbekistan		
				Vietnam		
				Zimbabwe		
**Highest Stratum (LEB ≥ 71)**	Denmark	Australia	Australia	Argentina	Algeria	Algeria
	Iceland	Belarus	Austria	Australia	Argentina	Argentina
	Netherlands	Canada	Belarus	Austria	Australia	Armenia
	Norway	Denmark	Belgium	Barbados	Austria	Australia
	Sweden	Estonia	Bulgaria	Belarus	Barbados	Austria
		France	Canada	Belgium	Belgium	Bahamas
		Greece	Costa Rica	Belize	Belize	Barbados
		Iceland	Cuba	Bulgaria	Canada	Belgium
		Latvia	Denmark	Canada	Chile	Brazil
		Lithuania	Finland	Chile	Colombia	Bulgaria
		Netherlands	France	Costa Rica	Costa Rica	Canada
		New Zealand	Germany	Croatia	Croatia	Chile
		Norway	Greece	Cuba	Cuba	Colombia
		Sweden	Iceland	Denmark	Denmark	Costa Rica
		Switzerland	Ireland	Finland	Ecuador	Croatia
		United Kingdom	Israel	France	El Salvador	Cuba
			Italy	Germany	Finland	Denmark
			Japan	Greece	France	Ecuador
			Lithuania	Iceland	Georgia	El Salvador
			Luxembourg	Ireland	Germany	Estonia
			Malta	Israel	Greece	Finland
			Netherlands	Italy	Iceland	France
			New Zealand	Jamaica	Ireland	Georgia
			Norway	Japan	Israel	Germany
			Panama	Lithuania	Italy	Greece

	Year 1955	Year 1965	Year 1975	Year 1985	Year 1995	Year 2005
			Spain	Luxembourg	Jamaica	Hungary
			Sweden	Malta	Japan	Iceland
			Switzerland	Netherlands	Lebanon	Iran
			United Kingdom	New Zealand	Luxembourg	Ireland
			United States	Norway	Malaysia	Israel
				Panama	Malta	Italy
				Paraguay	Mexico	Jamaica
				Portugal	Netherlands	Japan
				Qatar	New Zealand	Lebanon
				Serbia	Nicaragua	Lithuania
				Singapore	Norway	Luxembourg
				Slovenia	Oman	Malaysia
				Spain	Panama	Maldives
				Sweden	Paraguay	Malta
				Switzerland	Peru	Mauritius
				United Kingdom	Poland	Mexico
				United States	Portugal	Morocco
				Uruguay	Qatar	Netherlands
					Serbia	New Zealand
					Singapore	Nicaragua
					Slovak Republic	Norway
					Slovenia	Oman
					Spain	Panama
					Sri Lanka	Paraguay
					Sweden	Peru
					Switzerland	Poland
					Tunisia	Portugal
					United Kingdom	Qatar
					United States	Romania
					Uruguay	Serbia
						Singapore
						Slovak Republic
						Slovenia
						Spain
						Sri Lanka
						Sweden
						Switzerland
						Thailand
						Tunisia
						Turkey
						United Kingdom
						United States
						Uruguay
						Vietnam

Table A-2-4: Summary statistics, Lowest Stratum (LEB < 51)

Variables	Decade 1950-59		Decade 1960-69		Decade 1970-79		Decade 1980-89		Decade 1990-99		Decade 2000-09	
	**Mean (SD)**	**Obs.**	**Mean (SD)**	**Obs.**	**Mean (SD)**	**Obs.**	**Mean (SD)**	**Obs.**	**Mean (SD)**	**Obs.**	**Mean (SD)**	**Obs.**
Life Expectancy at Birth	42.76	635	45.95	435	47.43	197	49.02	106	47.85	82	47.70	60
	(4.09)		(3.32)		(2.62)		(1.83)		(4.83)		(2.12)	
LEB decade gain	7.38	635	5.26	435	3.92	197	2.73	106	-2.56	82	-6.82	60
	(3.59)		(1.96)		(2.54)		(2.40)		(6.02)		(6.64)	
GDP per capita, 2005, PPP $	1841	172	1275	348	950	187	814	98	850	82	1575	60
	(1079)		(1164)		(523)		(437)		(588)		(1953)	
Population density per km^2	33.35	635	46.71	435	55.56	197	59.58	106	82.98	82	70.03	60
	(56.99)		(78.16)		(73.45)		(74.75)		(90.03)		(63.38)	
CO2 in tons per capita	0.28	421	0.22	393	0.21	187	0.16	98	0.16	82	0.54	53
	(0.65)		(0.32)		(0.27)		(0.15)		(0.20)		(0.61)	
Total Fertility Rate	6.51	635	6.62	435	6.76	197	7.17	106	6.73	82	4.85	60
	(0.65)		(0.63)		(0.73)		(0.54)		(0.65)		(1.25)	
HIV Prevalence	0.00	635	0.00	435	0.45	197	1.88	106	6.04	82	17.03	60
	(0.00)		(0.00)		(0.85)		(2.39)		(5.34)		(7.83)	

Table A-2-5: Summary statistics, Stratum II (51 ≤ LEB < 61)

Variables	Decade 1950-59		Decade 1960-69		Decade 1970-79		Decade 1980-89		Decade 1990-99		Decade 2000-09	
	**Mean (SD)**	**Obs.**	**Mean (SD)**	**Obs.**	**Mean (SD)**	**Obs.**	**Mean (SD)**	**Obs.**	**Mean (SD)**	**Obs.**	**Mean (SD)**	**Obs.**
Life Expectancy at Birth	56.83	320	55.73	316	55.91	414	56.33	360	56.28	333	56.30	297
	(2.83)		(3.00)		(2.94)		(2.79)		(3.08)		(2.67)	
LEB decade gain	12.76	320	5.97	316	5.27	414	3.15	360	0.72	333	1.89	297
	(7.76)		(1.73)		(2.27)		(2.55)		(3.41)		(5.10)	
GDP per capita, 2005, PPP $	2353	127	2316	228	2410	406	1703	358	1615	331	2276	297
	(1393)		(1595)		(2591)		(1936)		(2090)		(3039)	
Population density per km^2	69.36	320	33.91	316	50.26	414	68.44	360	67.78	333	60.98	297
	(190.49)		(56.38)		(97.47)		(122.50)		(106.84)		(80.17)	
CO2 in tons per capita	1.50	206	0.91	237	1.09	384	0.66	346	0.51	318	0.86	297
	(2.58)		(1.30)		(1.90)		(1.35)		(0.92)		(2.01)	
Total Fertility Rate	5.53	320	6.50	316	6.58	414	6.28	360	5.66	333	5.46	297
	(1.35)		(0.84)		(0.71)		(0.70)		(1.01)		(1.14)	
HIV Prevalence	0.00	320	0.00	316	0.49	414	1.45	360	3.46	333	4.59	297
	(0.00)		(0.00)		(1.14)		(2.23)		(5.38)		(5.00)	

Table A-2-6: Summary statistics, Stratum III ($61 \leq LEB < 71$)

Variables	Decade 1950-59		Decade 1960-69		Decade 1970-79		Decade 1980-89		Decade 1990-99		Decade 2000-09	
	**Mean (SD)**	**Obs.**	**Mean (SD)**	**Obs.**	**Mean (SD)**	**Obs.**	**Mean (SD)**	**Obs.**	**Mean (SD)**	**Obs.**	**Mean (SD)**	**Obs.**
Life Expectancy at Birth	66.11 (2.75)	384	66.84 (3.13)	494	66.71 (2.87)	488	66.93 (2.85)	492	66.97 (2.98)	425	66.42 (3.12)	340
LEB decade gain	11.55 (9.55)	384	4.35 (2.14)	494	3.30 (2.93)	488	3.42 (2.76)	492	1.75 (2.89)	425	2.42 (2.81)	340
GDP per capita, 2005, PPP $	8123 (4131)	219	7548 (5420)	275	6750 (5598)	338	5531 (4800)	368	4922 (4572)	396	4363 (4530)	340
Population density per km^2	139.10 (295.44)	384	161.22 (400.42)	494	135.95 (416.39)	488	81.76 (112.27)	492	103.53 (164.34)	425	114.69 (177.81)	340
CO2 in tons per capita	4.97 (6.12)	313	6.21 (12.27)	396	6.14 (11.97)	363	3.49 (4.53)	376	3.57 (3.88)	399	2.72 (3.89)	340
Total Fertility Rate	3.22 (1.20)	384	3.82 (1.62)	494	3.89 (1.57)	488	3.99 (1.54)	492	3.22 (1.37)	425	3.17 (1.26)	340
HIV Prevalence	0.00 (0.00)	384	0.00 (0.00)	494	0.13 (0.38)	488	0.86 (2.45)	492	0.82 (2.09)	425	0.78 (2.00)	338

Table A-2-7: Summary statistics, Highest Stratum (LEB ≥ 71)

Variables	Decade 1950-59		Decade 1960-69		Decade 1970-79		Decade 1980-89		Decade 1990-99		Decade 2000-09	
	Mean (SD)	Obs.	Mean (SD)	Obs.	Mean (SD)	Obs.	Mean (SD)	Obs.	Mean (SD)	Obs.	Mean (SD)	Obs.
Life Expectancy at Birth	72.31	51	72.25	145	72.92	291	74.19	432	75.23	550	76.44	693
	(0.81)		(0.96)		(1.39)		(1.93)		(2.51)		(3.08)	
LEB decade gain	4.80	51	2.43	145	2.08	291	2.52	432	2.32	550	2.39	693
	(2.69)		(1.85)		(1.51)		(1.11)		(1.70)		(1.16)	
GDP per capita, 2005, PPP $	11835	51	15343	117	16854	269	17820	383	17931	549	20067	693
	(2013)		(3878)		(6579)		(9289)		(12379)		(16291)	
Population density per km^2	74.69	51	77.48	145	151.83	291	220.99	432	224.20	550	241.23	693
	(98.10)		(88.75)		(327.03)		(601.16)		(684.10)		(769.11)	
CO2 in tons per capita	4.99	51	7.19	122	9.89	273	8.44	390	7.50	543	7.01	693
	(1.23)		(2.98)		(8.52)		(7.34)		(8.34)		(7.60)	
Total Fertility Rate	2.95	51	2.77	145	2.39	291	2.23	432	2.23	550	1.93	693
	(0.62)		(0.56)		(0.86)		(0.90)		(0.89)		(0.54)	
HIV Prevalence	0.00	51	0.00	145	0.04	291	0.14	432	0.24	550	0.33	690
	(0.00)		(0.00)		(0.07)		(0.28)		(0.38)		(0.44)	

Table A-2-8: F-tests of equality between decadal dummies parameters, 1960-69 against 2000-09 and 1980-89 against 2000-09

	**All data (1950-2009)** [(1)]			**IHME data (1970-2009)** [(2)]		
	Parameter Decade 1960-69	Parameter Decade 2000-09	F-statistic (p-value)	Parameter Decade 1980-89	Parameter Decade 2000-09	F-statistic (p-value)
Lowest Stratum (LEB < 51)	-4.1708	-8.4908	30.6527	-1.8770	-3.2594	2.9265
			(0.0000)			(0.0880)
Stratum II (51 ≤ LEB < 61)	-4.7990	-11.4248	188.2726	-2.5990	-2.8321	0.5248
			(0.0000)			(0.4689)
Stratum III (61 ≤ LEB < 71)	-4.4669	-7.2831	35.2863	-0.7280	-1.4739	9.3644
			(0.0000)			(0.0023)
Highest Stratum (LEB ≥ 71)	-2.9610	-1.8926	23.1736	0.2793	-0.0444	7.0894
			(0.0000)			(0.0078)

Note:
(1) The F-tests comparing the coefficient on 1960-1969 to 2000-2009 (with 1950-59 as reference stratum)
(2) The F-tests comparing the coefficient on 1980-1989 to 2000-2009 (with 1970-79 as reference stratum)

Table A-2-9: The effects of LEB, income per capita, fertility, population density, CO2 emissions, and time on LEB decadal gains, 1950 – 2009, Fixed effects model

	LEB decadal gains				
	**Lowest Stratum (LEB < 51)**	**Stratum II (51 ≤ LEB < 61)**	**Stratum III (61 ≤ LEB < 71)**	**Highest Stratum (LEB ≥ 71)**	**Total**
Life Expectancy at Birth	2.279***	1.618***	0.594***	0.716***	3.604***
	(0.118)	(0.0819)	(0.0777)	(0.0552)	(0.0718)
GDP per capita, 2005, PPP $	0.248***	0.158**	0.0652	-0.0579	-0.108***
	(0.0832)	(0.0623)	(0.0654)	(0.0419)	(0.0382)
Population density per km^2	-1.329***	-1.319***	-0.937***	-0.490***	-1.529***
	(0.142)	(0.140)	(0.0811)	(0.0409)	(0.0903)
CO2 in tons per capita	-0.258***	-0.0880	-0.145**	-0.201***	-0.145***
	(0.0913)	(0.0622)	(0.0569)	(0.0251)	(0.0367)
Total Fertility Rate	0.0966	-0.214***	0.219***	0.262***	-0.000115
	(0.0834)	(0.0818)	(0.0729)	(0.0313)	(0.0495)
Decade 1960-69	-3.181***	-4.345***	-5.212***	-2.968***	-5.545***
	(0.322)	(0.403)	(0.248)	(0.181)	(0.138)
Decade 1970-79	-5.003***	-6.739***	-5.597***	-2.098***	-7.545***
	(0.429)	(0.487)	(0.327)	(0.179)	(0.174)
Decade 1980-89	-6.738***	-9.671***	-6.686***	-1.899***	-9.589***
	(0.515)	(0.537)	(0.384)	(0.184)	(0.215)
Decade 1990-99	-8.862***	-11.26***	-7.440***	-2.599***	-11.01***
	(0.582)	(0.589)	(0.430)	(0.200)	(0.258)
Decade 2000-09	-5.328***	-10.01***	-6.971***	-2.721***	-10.84***
	(0.805)	(0.665)	(0.493)	(0.232)	(0.306)
Constant	7.876***	12.01***	9.509***	4.842***	11.36***
	(0.327)	(0.487)	(0.330)	(0.193)	(0.179)
Observations	1,019	1,819	2,425	2,527	7,827
R-squared	0.470	0.474	0.388	0.305	0.471
Number of countries	62	91	128	94	173
Country FE	Yes	Yes	Yes	Yes	Yes

Standard errors in parentheses

*** P<0.01, ** P<0.05, * P<0.1

Table A-2-10: LEB decade gains, by region, decade and LEB strata

Decade	Lowest Stratum (LEB < 51)		Stratum II 51 ≤ LEB < 61		Stratum III 61 ≤ LEB < 71		Highest Stratum (LEB ≥ 71)	
	Mean (SD) (N)	IQR	Mean (SD) (N)	IQR	Mean (SD) (N)	IQR	Mean (SD) (N)	IQR
**East Asia and Pacific**								
1950-1959	9.06 (3.64) (42)	6.93 - 11.76	13.88 (6.61) (53)	9.13 - 17.72	9.22 (9.06) (33)	2.80 - 13.78	2.24 (0.24) (2)	2.06 - 2.41
1960-1969	5.48 (2.72) (26)	2.60 - 7.63	6.59 (1.85) (36)	5.51 - 8.08	4.90 (1.68) (47)	3.82 - 5.88	2.01 (1.69) (21)	0.83 - 2.00
1970-1979	0.93 (1.43) (8)	1.15 - 1.64	5.45 (3.56) (36)	4.56 - 7.39	3.61 (1.63) (54)	2.43 - 5.01	2.07 (1.54) (32)	0.64 - 3.61
1980-1989	. (.) (0)	. - .	3.33 (3.97) (26)	-0.80 - 6.90	3.29 (2.42) (57)	1.70 - 3.80	3.01 (0.67) (47)	2.60 - 3.50
1990-1999	. (.) (0)	. - .	1.40 (1.89) (23)	-0.10 - 3.40	1.80 (1.65) (47)	-0.20 - 3.40	2.78 (0.81) (60)	2.40 - 3.30
2000-2009	. (.) (0)	. - .	1.30 (0.34) (11)	1.00 - 1.60	1.81 (1.70) (49)	0.40 - 2.30	2.47 (0.96) (70)	2.00 - 3.10
**Europe and Central Asia**								
1950-1959	10.58 (6.40) (13)	6.79 - 10.20	18.11 (10.20) (96)	8.27 - 26.02	13.42 (10.47) (242)	6.19 - 17.24	4.91 (2.69) (49)	3.14 - 6.20
1960-1969	6.63 (0.50) (4)	6.25 - 7.01	4.23 (1.45) (44)	3.34 - 4.33	4.07 (2.24) (240)	2.49 - 5.08	2.46 (1.88) (112)	1.04 - 3.56
1970-1979	. (.) (0)	. - .	5.53 (1.80) (16)	3.83 - 7.26	1.96 (2.03) (196)	0.06 - 3.70	1.56 (1.06) (188)	0.99 - 2.27
1980-1989	.	. - .	.	. - .	1.75	-0.20 - 3.20	2.09	1.60 - 2.70

Decade	Lowest Stratum (LEB < 51)		Stratum II 51 ≤ LEB < 61		Stratum III 61 ≤ LEB < 71		Highest Stratum (LEB ≥ 71)	
	Mean (SD) (N)	IQR	Mean (SD) (N)	IQR	Mean (SD) (N)	IQR	Mean (SD) (N)	IQR
	(.)		(.)		(2.05)		(0.91)	
	(0)		(0)		(159)		(241)	
1990-1999	.	. - .	-4.90	-4.90 - -4.90	-0.38	-1.60 - 0.60	1.82	1.40 - 2.40
	(.)		(.)		(2.00)		(0.75)	
	(0)		(1)		(154)		(245)	
2000-2009	.	. - .	.	. - .	1.40	0.00 - 3.00	2.43	2.00 - 2.80
	(.)		(.)		(2.31)		(0.84)	
	(0)		(0)		(90)		(310)	
**Latin America and the Caribbean**								
1950-1959	6.80	5.34 - 7.69	8.54	6.70 - 10.53	7.24	5.13 - 9.72	.	. - .
	(1.83)		(2.40)		(3.43)		(.)	
	(71)		(121)		(58)		(0)	
1960-1969	5.09	4.07 - 5.82	6.39	5.11 - 7.49	4.63	2.56 - 6.05	6.62	6.60 - 6.65
	(0.88)		(1.81)		(1.91)		(0.03)	
	(42)		(63)		(143)		(2)	
1970-1979	4.20	3.90 - 4.64	6.58	5.51 - 7.76	4.30	2.30 - 5.21	5.07	3.77 - 5.78
	(0.48)		(1.95)		(3.19)		(1.22)	
	(11)		(33)		(176)		(30)	
1980-1989	3.60	3.60 - 3.60	4.92	4.30 - 6.10	3.91	2.30 - 4.70	3.00	2.55 - 3.85
	(.)		(1.86)		(2.22)		(1.29)	
	(1)		(21)		(140)		(88)	
1990-1999	.	. - .	2.86	2.50 - 3.20	2.32	0.90 - 3.80	2.07	0.80 - 3.10
	(.)		(0.57)		(2.43)		(1.69)	
	(0)		(10)		(94)		(146)	
2000-2009	.	. - .	4.40	3.90 - 4.70	1.32	-0.70 - 3.70	2.03	1.60 - 2.70
	(.)		(0.36)		(2.58)		(1.20)	
	(0)		(7)		(70)		(173)	
**Middle East and North Africa**								
1950-1959	9.74	7.07 - 12.62	14.17	9.07 - 19.04	12.49	6.33 - 18.10	.	. - .
	(3.43)		(6.00)		(7.85)		(.)	
	(67)		(12)		(31)		(0)	

Decade	Lowest Stratum (LEB < 51)		Stratum II 51 ≤ LEB < 61		Stratum III 61 ≤ LEB < 71		Highest Stratum (LEB ≥ 71)	
	Mean (SD) (N)	IQR	Mean (SD) (N)	IQR	Mean (SD) (N)	IQR	Mean (SD) (N)	IQR
1960-1969	7.41	6.36 - 9.09	6.16	5.13 - 6.45	4.48	2.95 - 6.08	.	. - .
	(1.56)		(1.31)		(2.08)		(.)	
	(25)		(45)		(40)		(0)	
1970-1979	-19.81	-19.81 - -19.81	6.28	4.56 - 8.28	5.14	3.06 - 9.66	2.51	2.18 - 2.68
	(.)		(2.25)		(5.16)		(0.60)	
	(1)		(58)		(29)		(22)	
1980-1989	.	. - .	2.09	0.05 - 6.30	6.24	5.20 - 8.80	3.52	2.75 - 4.35
	(.)		(4.61)		(4.17)		(1.15)	
	(0)		(20)		(58)		(32)	
1990-1999	.	. - .	-0.73	-0.90 - -0.50	5.71	4.80 - 6.30	4.46	2.60 - 5.00
	(.)		(0.24)		(1.60)		(2.86)	
	(0)		(10)		(33)		(67)	
2000-2009	.	. - .	-0.06	-0.45 - 0.30	2.61	1.80 - 3.30	2.72	2.10 - 2.90
	(.)		(0.57)		(0.94)		(1.28)	
	(0)		(8)		(12)		(90)	
**North America**								
1950-1959	.	. - .	.	. - .	3.76	3.22 - 4.41	.	. - .
	(.)		(.)		(0.78)		(.)	
	(0)		(0)		(20)		(0)	
1960-1969	.	. - .	.	. - .	1.13	0.68 - 1.73	2.11	1.73 - 2.54
	(.)		(.)		(0.54)		(0.43)	
	(0)		(0)		(10)		(10)	
1970-1979	.	. - .	.	. - .	0.97	0.97 - 0.97	2.05	1.64 - 2.39
	(.)		(.)		(.)		(0.62)	
	(0)		(0)		(1)		(19)	
1980-1989	.	. - .	.	. - .	.	. - .	2.57	2.30 - 3.00
	(.)		(.)		(.)		(0.52)	
	(0)		(0)		(0)		(20)	
1990-1999	.	. - .	.	. - .	.	. - .	1.67	1.45 - 1.90
	(.)		(.)		(.)		(0.36)	
	(0)		(0)		(0)		(20)	

Decade	Lowest Stratum (LEB < 51)		Stratum II 51 ≤ LEB < 61		Stratum III 61 ≤ LEB < 71		Highest Stratum (LEB ≥ 71)	
	Mean (SD) (N)	IQR	Mean (SD) (N)	IQR	Mean (SD) (N)	IQR	Mean (SD) (N)	IQR
2000-2009	.	. - .	.	. - .	.	. - .	1.75	1.50 - 1.95
	(.)		(.)		(.)		(0.33)	
	(0)		(0)		(0)		(20)	
**South Asia**								
1950-1959	5.62	3.26 - 6.17	8.95	6.28 - 11.90	.	. - .	.	. - .
	(4.83)		(3.29)		(.)		(.)	
	(60)		(10)		(0)		(0)	
1960-1969	6.32	5.21 - 7.26	8.33	7.37 - 9.24	5.34	4.97 - 5.64	.	. - .
	(1.97)		(1.09)		(0.52)		(.)	
	(54)		(8)		(8)		(0)	
1970-1979	5.35	4.47 - 6.58	6.21	4.87 - 7.11	3.33	2.42 - 4.06	.	. - .
	(1.64)		(1.86)		(1.04)		(.)	
	(23)		(37)		(10)		(0)	
1980-1989	.	. - .	5.01	4.35 - 5.40	5.10	4.80 - 5.60	3.77	2.85 - 4.70
	(.)		(1.06)		(0.51)		(1.18)	
	(0)		(48)		(18)		(4)	
1990-1999	.	. - .	4.71	3.40 - 6.30	5.18	3.70 - 6.70	2.59	1.15 - 2.90
	(.)		(1.38)		(2.31)		(2.20)	
	(0)		(21)		(37)		(12)	
2000-2009	.	. - .	.	. - .	4.13	3.50 - 5.40	4.20	1.65 - 6.75
	(.)		(.)		(1.94)		(2.75)	
	(0)		(0)		(50)		(20)	
**Sub-Saharan Africa**								
1950-1959	7.06	4.73 - 8.83	11.34	7.18 - 15.29	.	. - .	.	. - .
	(3.19)		(5.05)		(.)		(.)	
	(382)		(28)		(0)		(0)	
1960-1969	4.86	3.59 - 6.02	5.97	4.94 - 7.04	7.78	6.32 - 9.29	.	. - .
	(1.84)		(1.43)		(1.81)		(.)	
	(284)		(120)		(6)		(0)	
1970-1979	4.00	2.25 - 5.47	4.64	3.40 - 5.67	4.10	2.54 - 5.55	.	. - .
	(1.82)		(1.91)		(1.48)		(.)	

**Decade**	**Lowest Stratum (LEB < 51)**		**Stratum II 51 ≤ LEB < 61**		**Stratum III 61 ≤ LEB < 71**		**Highest Stratum (LEB ≥ 71)**	
	Mean (SD) (N)	IQR	Mean (SD) (N)	IQR	Mean (SD) (N)	IQR	Mean (SD) (N)	IQR
	(154)		(234)		(22)		(0)	
1980-1989	2.72	1.40 - 4.60	2.70	2.00 - 3.90	3.59	2.80 - 4.20	.	. - .
	(2.41)		(2.14)		(1.16)		(.)	
	(105)		(245)		(60)		(0)	
1990-1999	-2.56	-3.90 - 1.40	0.34	-1.50 - 2.75	2.03	1.35 - 2.90	.	. - .
	(6.02)		(3.50)		(2.22)		(.)	
	(82)		(268)		(60)		(0)	
2000-2009	-6.82	-12.95 - -1.05	1.91	-0.30 - 4.40	4.02	2.70 - 4.30	1.69	1.50 - 1.90
	(6.64)		(5.32)		(3.61)		(0.22)	
	(60)		(271)		(69)		(10)	

3. Figures

Figure A-3-1: LEB decadal gains by decade, Stratum II (51≤LEB<61) and Stratum III (61≤LEB<71)

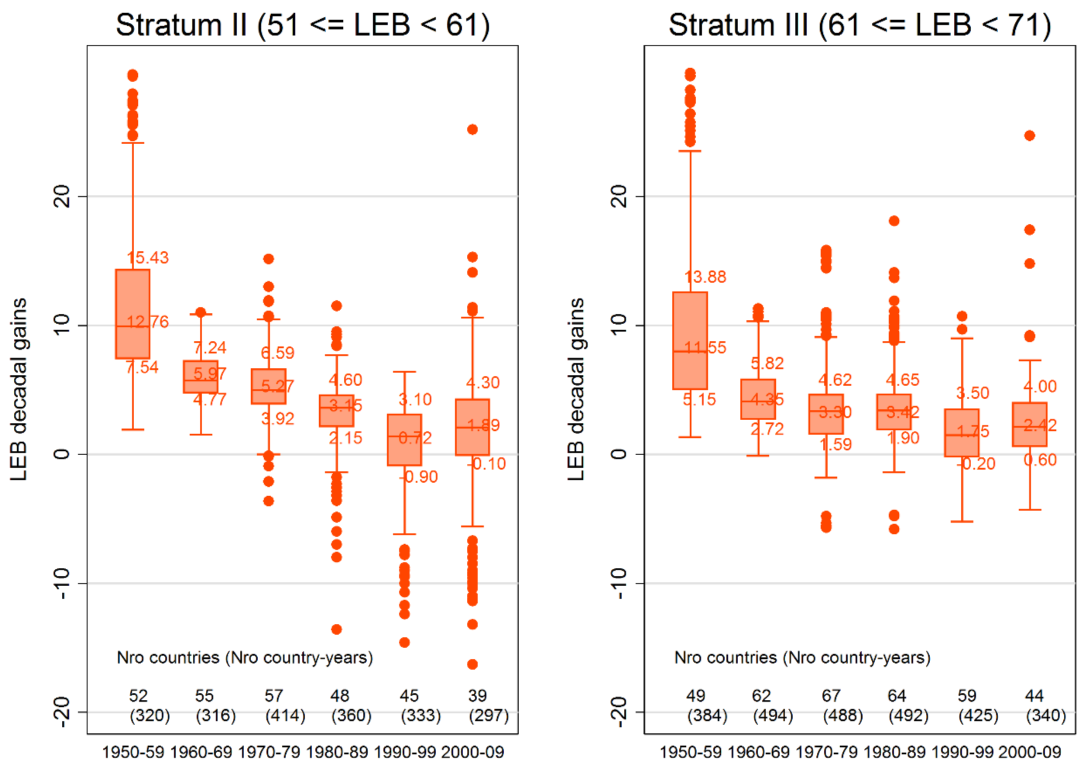

Figure A-3-2: LEB decadal gains distribution by strata, comparison between decades

Lowest Stratum (LEB < 51)

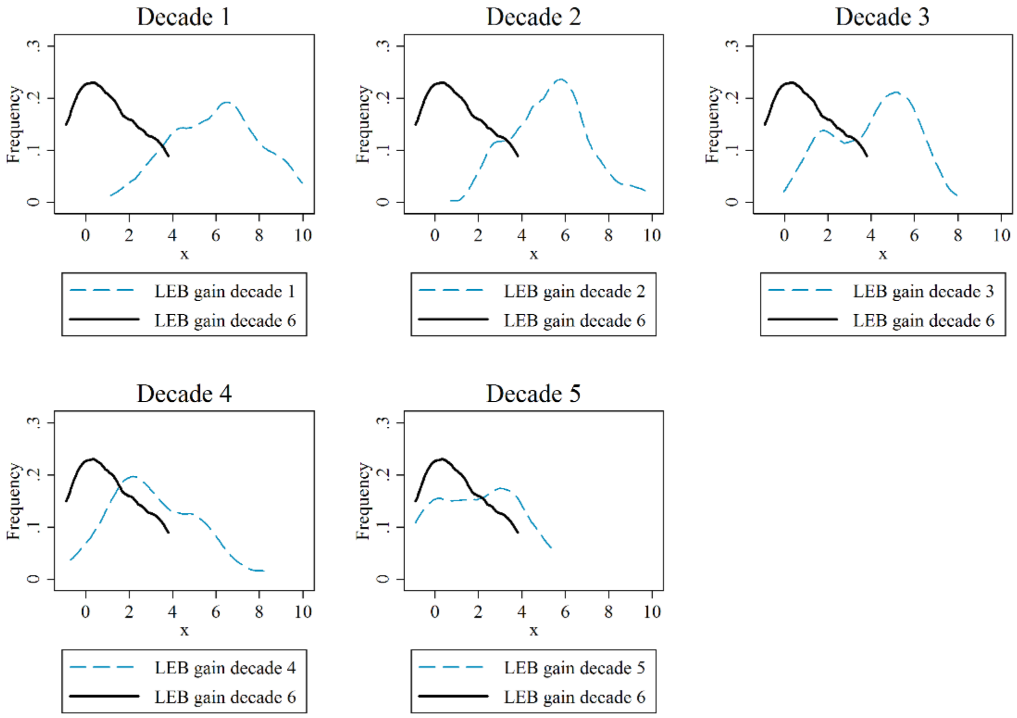

Stratum II (51 ≤ LEB < 61)

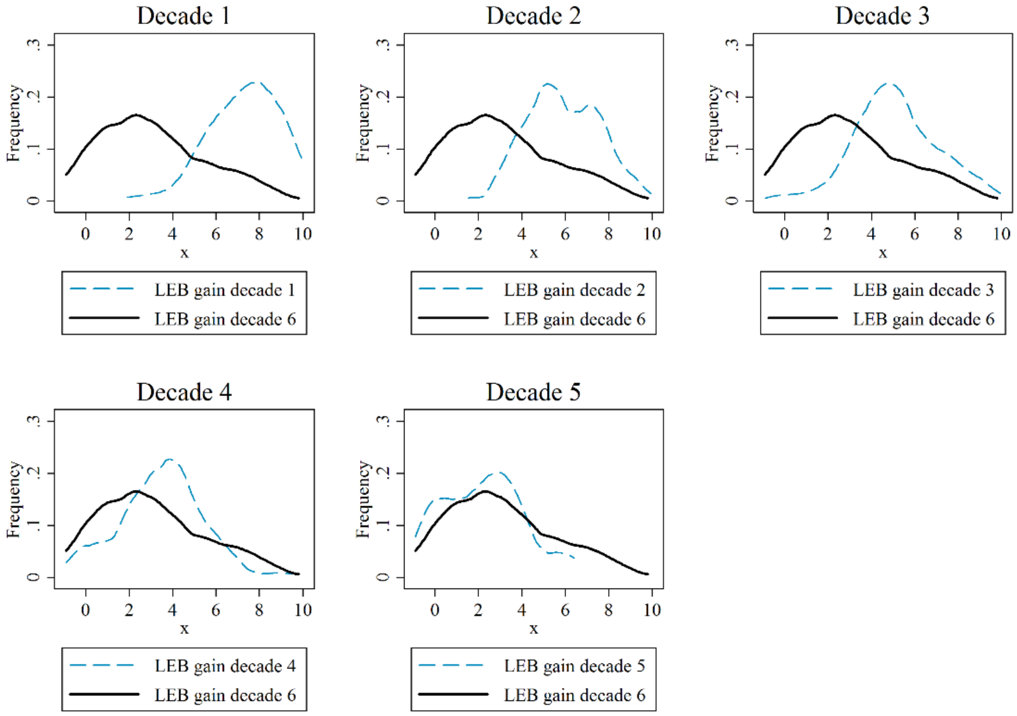

Stratum III (61 ≤ LEB < 71)

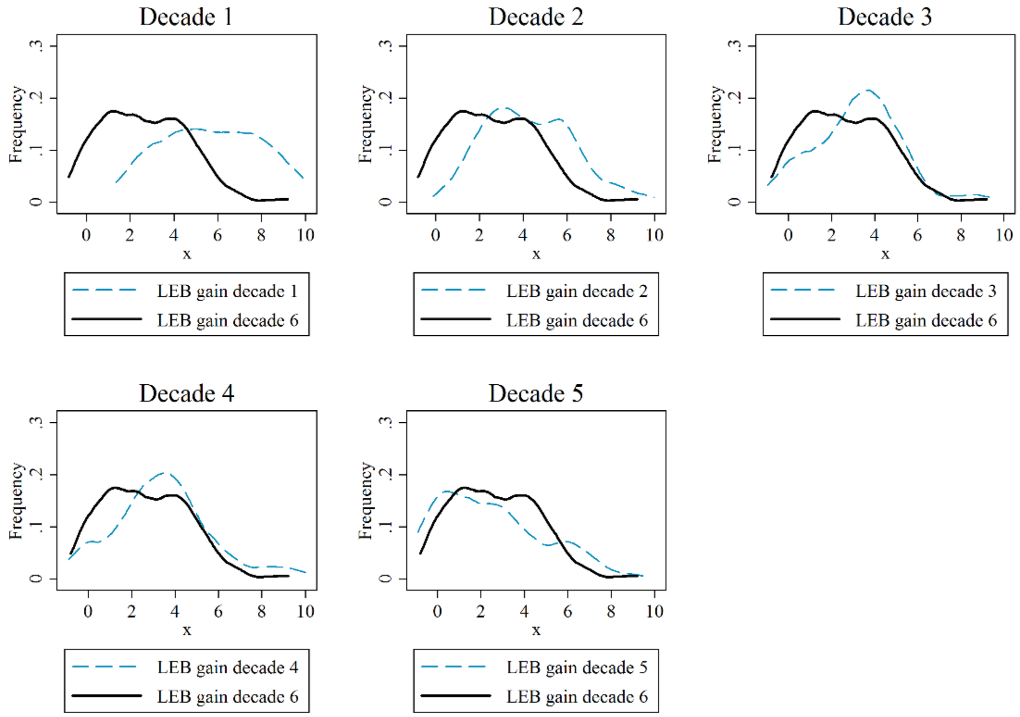

Highest Stratum (LEB ≥ 71)

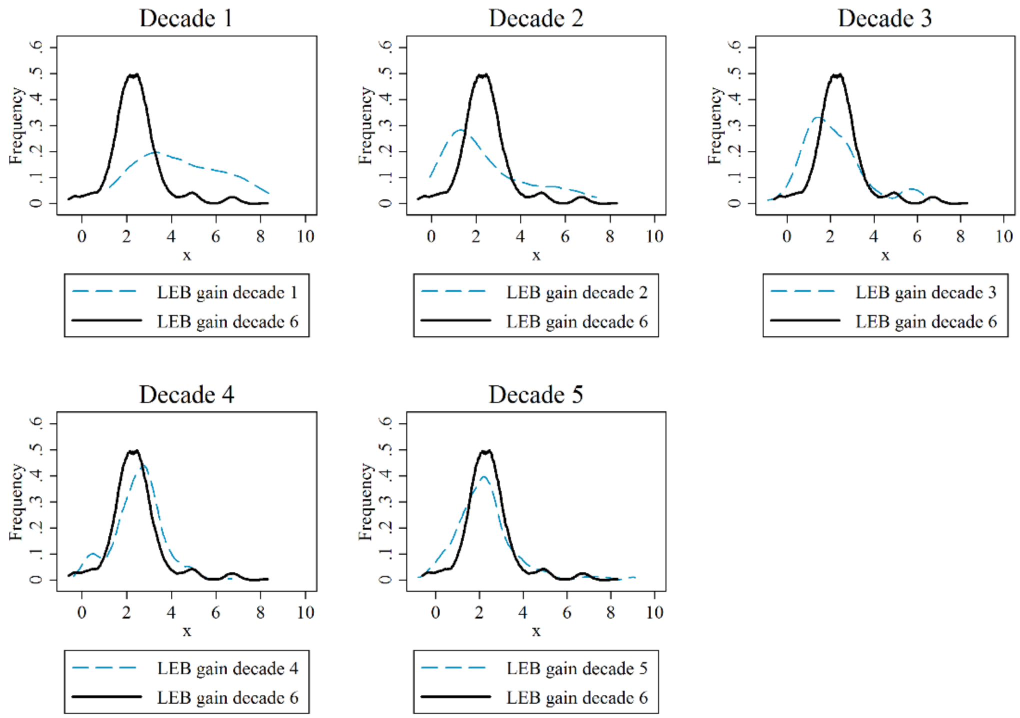

4. HIV imputation

The global HIV/AIDS pandemic had a high impact on the overall number of deaths worldwide. In low income countries an estimated 1–4 years of life expectancy are lost due to HIV/AIDS in those older than 5 years [14]. For years prior to 1970, it was assumed HIV prevalence was virtually zero, since the disease was acknowledged in 1981 [15]. For countries lacking complete HIV data for years 1970-1989, country-specific trends were used to back-cast HIV rates.

Back-cast imputations were performed using a Fixed Effects (FE) model for each country following a cubic function of time, where HIV prevalence was used as the dependent variable. HIV prevalence was imputed for 1970 – 1989, based on information from 1990 – 2009. In order to extrapolate these values, countries had to have at least 18 observations in the 20 year-period. The general equation for the fixed effects regressions was $E[HIV]_{it} = \beta_0 + \alpha_1{*}year + \alpha_2{*}year^2 + \alpha_3{*}year^3 + \mu_i + \varepsilon_{it}$. Table A-4-1 lists the countries that followed this imputation (130 countries, imputation A).

Some countries have maintained the same HIV prevalence between 1990 – 2009, making a FE regression not possible. For these countries, the assumption was they had the same constant HIV prevalence as in 1990 – 2009. Table A-4-1 lists the countries that followed this imputation (16 countries, imputation B).

Table A-4-1: Countries with HIV imputed values, FE regression (imputation A) and constant prevalence (imputation B)

Imputation	Country	ISO Code	Imputation	Country	ISO Code
A	Angola	AGO	A	Latvia	LVA
A	Argentina	ARG	A	Morocco	MAR
A	Armenia	ARM	A	Moldova	MDA
A	Australia	AUS	A	Madagascar	MDG
A	Austria	AUT	A	Mexico	MEX
A	Azerbaijan	AZE	A	Mali	MLI
A	Burundi	BDI	A	Malta	MLT
A	Belgium	BEL	A	Myanmar	MMR
A	Benin	BEN	A	Mozambique	MOZ
A	Burkina Faso	BFA	A	Mauritania	MRT
A	Bulgaria	BGR	A	Mauritius	MUS
A	Bahamas	BHS	A	Malawi	MWI
A	Belarus	BLR	A	Malaysia	MYS
A	Belize	BLZ	A	Namibia	NAM
A	Bolivia	BOL	A	Niger	NER
A	Brazil	BRA	A	Nigeria	NGA
A	Barbados	BRB	A	Nicaragua	NIC
A	Bhutan	BTN	A	Netherlands	NLD
A	Botswana	BWA	A	Norway	NOR
A	Central African Rep.	CAF	A	Nepal	NPL
A	Canada	CAN	A	New Zealand	NZL
A	Switzerland	CHE	A	Oman	OMN
A	Chile	CHL	A	Pakistan	PAK
A	Cote d'Ivoire	CIV	A	Panama	PAN
A	Cameroon	CMR	A	Peru	PER
A	Congo, Rep.	COG	A	Papua New Guinea	PNG
A	Colombia	COL	A	Poland	POL
A	Comoros	COM	A	Portugal	PRT
A	Costa Rica	CRI	A	Paraguay	PRY
A	Cuba	CUB	A	Romania	ROM
A	Djibouti	DJI	A	Russia	RUS
A	Denmark	DNK	A	Rwanda	RWA
A	Dominican Rep.	DOM	A	Sudan	SDN

Imputation	Country	ISO Code	Imputation	Country	ISO Code
A	Algeria	DZA	A	Senegal	SEN
A	Ecuador	ECU	A	Singapore	SGP
A	Eritrea	ERI	A	Sierra Leone	SLE
A	Spain	ESP	A	El Salvador	SLV
A	Estonia	EST	A	Somalia	SOM
A	Finland	FIN	A	Serbia	SRB
A	Fiji	FJI	A	Suriname	SUR
A	France	FRA	A	Sweden	SWE
A	Gabon	GAB	A	Swaziland	SWZ
A	United Kingdom	GBR	A	Chad	TCD
A	Georgia	GEO	A	Togo	TGO
A	Ghana	GHA	A	Thailand	THA
A	Guinea	GIN	A	Tajikistan	TJK
A	Gambia	GMB	A	Trinidad and Tobago	TTO
A	Guinea-Bissau	GNB	A	Tanzania	TZA
A	Equatorial Guinea	GNQ	A	Uganda	UGA
A	Greece	GRC	A	Ukraine	UKR
A	Guatemala	GTM	A	Uruguay	URY
A	Guyana	GUY	A	United States	USA
A	Honduras	HND	A	Uzbekistan	UZB
A	Haiti	HTI	A	Vietnam	VNM
A	Hungary	HUN	A	South Africa	ZAF
A	Indonesia	IDN	A	Zambia	ZMB
A	India	IND	A	Zimbabwe	ZWE
A	Ireland	IRL	B	Bangladesh	BGD
A	Iran	IRN	B	Czech Rep.	CZE
A	Iceland	ISL	B	Germany	DEU
A	Israel	ISR	B	Egypt	EGY
A	Italy	ITA	B	Croatia	HRV
A	Jamaica	JAM	B	Japan	JPN
A	Kazakhstan	KAZ	B	Korea, Rep.	KOR
A	Kenya	KEN	B	Sri Lanka	LKA
A	Kyrgyzstan	KGZ	B	Maldives	MDV
A	Cambodia	KHM	B	Mongolia	MNG
A	Laos	LAO	B	Philippines	PHL
A	Lebanon	LBN	B	Qatar	QAT
A	Liberia	LBR	B	Slovak Republic	SVK
A	Lesotho	LSO	B	Slovenia	SVN
A	Lithuania	LTU	B	Tunisia	TUN
A	Luxembourg	LUX	B	Turkey	TUR

References

1. Johansson K, Lindgren M. Documentation for Life Expectancy at birth (years) for countries and territories. 2014.

2. IHME. Life Expectancy at birth (years). Institute for Health Metrics and Evaluation. 2014. Available from: https://cloud.ihme.washington.edu/index.php/s/b89390325f728bbd99de0356d3be6900. [Accessed 04 January 2016].

3. WPP. World Population Prospects: The 2012 Revision - Life expectancy at birth, both sexes. United Nations Population Division. 2012.

4. HMD. Human Mortality Database. University of California, Berkeley and Max Planck Institute for Demographic Research. 2009. Available from: http://www.mortality.org or www.humanmortality.de. [Accessed 04 January 2016].

5. WPP. World Population Prospects: The 2006 Revision - Life expectancy at birth, both sexes. United Nations Population Division. 2008.

6. HMD. Human Mortality Database. University of California, Berkeley and Max Planck Institute for Demographic Research. 2013. Available from: http://www.mortality.org or www.humanmortality.de. [Accessed 04 January 2016].

7. HMD. Human Mortality Database. University of California, Berkeley and Max Planck Institute for Demographic Research. 2011. Available from: http://www.mortality.org or www.humanmortality.de. [Accessed 04 January 2016].

8. Heston, A., Summers, R., & Aten, B. Penn World Table Version 7.1, Center for International Comparisons of Production, Income and Prices at the University of Pennsylvania. 2012.

9. Ajus F, Lindgren M. Documentation for Children per Woman (Total Fertility Rate) for countries and territories. 2009.

10. WPP. World Population Prospects: The 2012 Revision - Children per women (total fertility) with projections. United Nations Population Division. 2013.

11. UN Population Division. World Population Prospects: The 2010 Revision - Population density (per square km). United Nations Population Division. 2013. Available from: http://esa.un.org/unpd/wpp/Excel-Data/population.htm. [Accessed 04 January 2016].

12. CDIAC. Per capita CO2 emissions (metric tons of CO2). Carbon Dioxide Information Analysis Center. 2015. Available from: http://cdiac.ornl.gov/. [Accessed 12 November 2015]. Carbon Dioxide Information Analysis Center;

13. UNAIDS. Estimated HIV Prevalence % - (Ages 15-49). United Nations Programme on HIV and AIDS. 2013. Available from: http://www.gapminder.org/gapminder-world/documentation/gd006. [Accessed 04 January 2016].

14. Jamison DT, Summers LH, Alleyne G, Arrow KJ, Berkley S, Binagwaho A, et al. Global health 2035: A world converging within a generation. Lancet. 2013;382:1898–955.

15. Hymes K, Greene J, Marcus A, William D, Cheung T, Prose N, et al. Kaposi'S Sarcoma in Homosexual Men—a Report of Eight Cases. Lancet. 1981;318:598–600.
